# Metabolic and tolerance engineering of *Komagataella phaffii* for 2-phenylethanol production through genome-wide scanning

**DOI:** 10.1186/s13068-024-02536-y

**Published:** 2024-07-22

**Authors:** Lijing Sun, Ying Gao, Renjie Sun, Ling Liu, Liangcai Lin, Cuiying Zhang

**Affiliations:** State Key Laboratory of Food Nutrition and Safety, Key Laboratory of Industrial Fermentation Microbiology, Ministry of Education, Tianjin Key Laboratory of Industrial Microbiology, College of Biotechnology, Tianjin University of Science and Technology, Tianjin, 300457 People’s Republic of China

**Keywords:** 2-Phenylethanol, *Komagataella phaffii*, Tolerance, Ehrlich pathway, Comparative transcriptome

## Abstract

**Background:**

2-Phenylethanol (2-PE) is one of the most widely used spices. Recently, 2-PE has also been considered a potential aviation fuel booster. However, the lack of scientific understanding of the 2-PE biosynthetic pathway and the cellular response to 2-PE cytotoxicity are the most important obstacles to the efficient biosynthesis of 2-PE.

**Results:**

Here, metabolic engineering and tolerance engineering strategies were used to improve the production of 2-PE in *Komagataella phaffii*. First, the endogenous genes encoding the amino acid permease *GAP1*, aminotransferase *AAT2*, phenylpyruvate decarboxylase *KDC2*, and aldehyde dehydrogenase *ALD4* involved in the Ehrlich pathway and the 2-PE stress response gene *NIT1* in *K. phaffii* were screened and characterized via comparative transcriptome analysis. Subsequently, metabolic engineering was employed to gradually reconstruct the 2-PE biosynthetic pathway, and the engineered strain S43 was obtained, which produced 2.98 g/L 2-PE in shake flask. Furthermore, transcriptional profiling analyses were utilized to screen for novel potential tolerance elements. Our results demonstrated that cells with knockout of the *PDR12* and *C4R2I5* genes exhibited a significant increase in 2-PE tolerance. To confirm the practical applications of these results, deletion of the *PDR12* and *C4R2I5* genes in the hyper 2-PE producing strain S43 dramatically increased the production of 2-PE by 18.12%, and the production was 3.54 g/L.

**Conclusion:**

This is the highest production of 2-PE produced by *K. phaffii* via l-phenylalanine conversion. These identified *K. phaffii* endogenous elements are highly conserved in other yeast species, suggesting that manipulation of these homologues might be a useful strategy for improving aromatic alcohol production. These results also enrich the understanding of aromatic compound biosynthetic pathways and 2-PE tolerance, and provide new elements and strategies for the synthesis of aromatic compounds by microbial cell factories.

**Supplementary Information:**

The online version contains supplementary material available at 10.1186/s13068-024-02536-y.

## Introduction

To date, many natural products and chemicals have been manufactured via fermentative production by efficient cell factories [1]. 2-Phenylethanol (2-PE) is a highly aromatic alcohol with a rose-like odor that is widely used in the cosmetics, pharmaceutical, and food industries [2]. Due to its high energy density and low moisture absorption, 2-PE is also used as a promising gasoline substitute [3, 4]. Furthermore, 2-PE serves as a building block for other products (styrene, phenylethyl acetate, and phenyl glycosides). The wide range of advantages of 2-PE has led to increasing product penetration in many industries across the globe, and the market size of 2-PE is expected to surpass USD 3.7 billion by 2028 (https://www.gminsights.com/industry-analysis/2-phenylethanol-market). Commercially 2-PE is produced mainly via chemical methods. However, chemically synthesized 2-PE derived from benzene compromises process safety and environmental sustainability. Additionally, the amount of 2-PE synthesized by chemical methods is limited in the food and cosmetic industries due to the presence of carcinogenic byproducts [5, 6]. Therefore, environmentally friendly and more sustainable approaches for the synthesis of 2-PE are urgently needed.

**Fig. 1:**
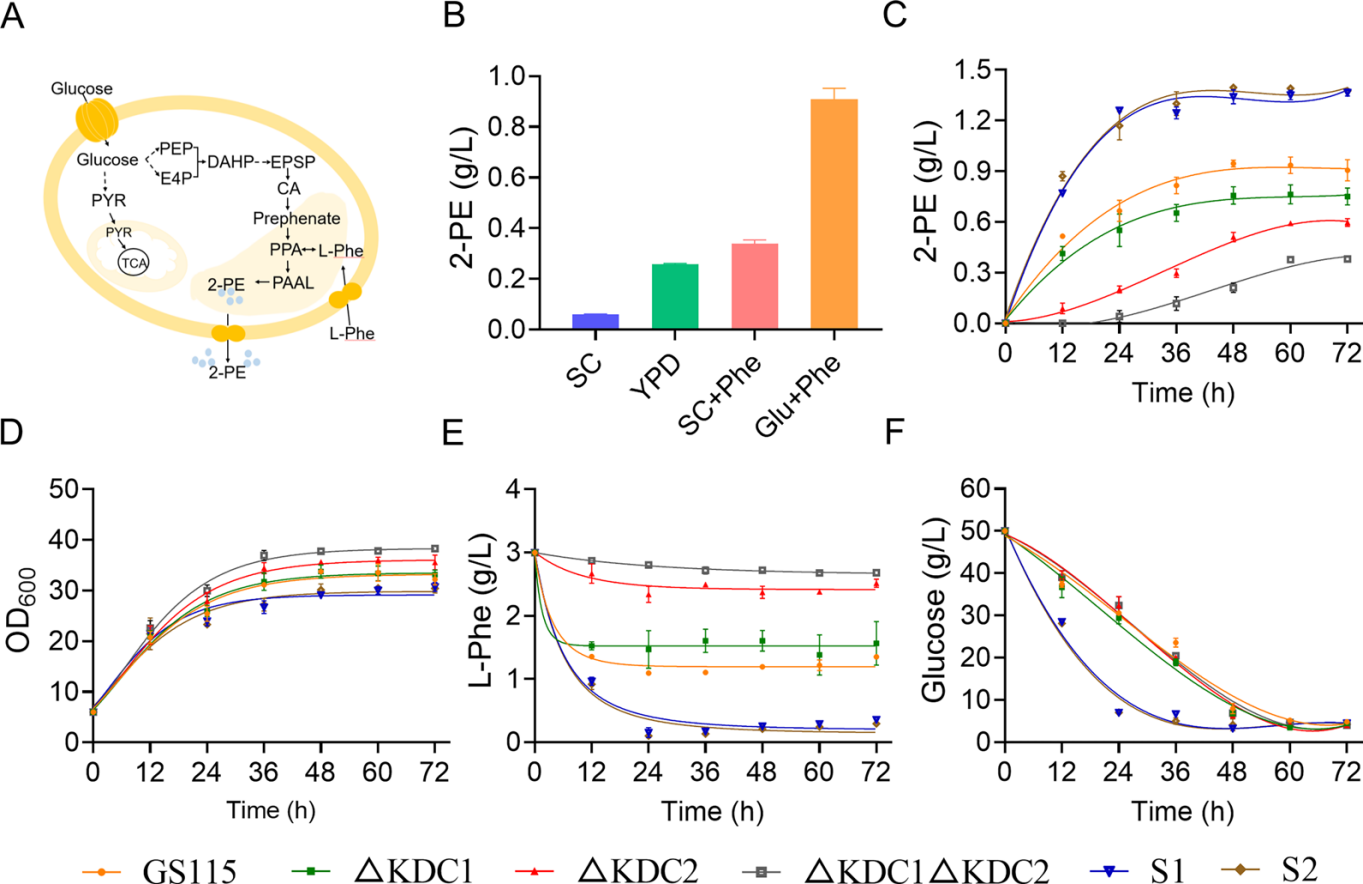
Identification of the phenylpyruvate decarboxylase gene in the Ehrlich pathway. **A** The 2-PE synthesis pathway in yeast. **B** Production of 2-PE by *K. phaffii* GS115 in different media. Defined synthetic complete (SC) medium: 6.7 g/L of yeast nitrogen base (YNB), 50 g/L glucose, when needed, 100 mg/L of histidine (His) was added. YPD: 10 g/L yeast extract, 20 g/L peptone, and 50 g/L glucose. In SC + Phe, 5 g/L of l-Phe is added into SC. Glu + Phe medium: 50 g/L glucose, 0.5 g/L MgSO_4_, 5 g/L KH_2_PO_4_, 5 g/L of l-Phe, 0.04 g/L of histidine (His). **C**–**F** Cell growth, 2-PE production, residual l-Phe, and residual glucose in parental strains and phenylpyruvate knockout and overexpression strains

The natural 2-PE biosynthetic pathway has been confirmed in many plants and microorganisms, such as *Saccharomyces cerevisiae* [7], *Yarrowia lipolytica* [8], *Bacillus licheniformis* [9], and *Enterobacter* sp*.* CGMCC 5087 [10]. To elevate 2-PE production, several strategies have been applied. The highest 6.3 g/L of 2-PE could be obtained with l-Phe as substrate in *S. cerevisiae* YS58 [11]. To the best of our knowledge (Table S1), this result represents the highest 2-PE production from the Ehrlich pathway in yeast. Moreover, and the *Yarrowia lipolytica* by applying genetic engineering method produced 2.43 g/L 2-PE, representing the highest production for de novo production of 2-PE in yeast [8]. Therefore, an efficient route for the synthesis of 2-PE is the conversion of l-phenylalanine (l-Phe) via the Ehrlich pathway (Fig. 1A) [12]. According to previous investigations, some elements involved in 2-PE conversion have been identified [13, 14]. For example, the gene *KDC1* (encoding phenylpyruvate decarboxylase) from *K. phaffii* was overexpressed in *E. coli*, and 2-PE production in the fermentation broth ranged from undetectable to 57 mg/L [15]. The *ARO10* gene (encoding phenylpyruvate decarboxylase) of *S. cerevisiae* was expressed in *K. phaffii*, and 2-PE was synthesized using methanol [16]. However, the use of *K. phaffii* as a chassis for the biosynthesis of 2-PE has not been systematically studied. Therefore, the important components involved in this pathway remain unknown.

In recent years, an increasing number of aromatic compounds have been found to be toxic, thus limiting their further production [17]. Lipophilic 2-PE can target the cell membrane for attack and increase membrane permeability to inhibit cell growth [18]. It can also affect cell growth by directly damaging the mitochondrial respiratory chain [19]. Therefore, the high concentration of 2-PE in the fermentation process has an adverse effect on cell growth and metabolism. Recent studies have shown that co-culture strategies are expected to increase 2-PE product yield [20–22], but the most direct way is to improve the tolerance of strains. However, a few studies on the tolerance of microorganisms to 2-PE have been performed, and the regulatory pathways and components have not been clearly revealed. As a highly tolerant 2-PE strain, *Bacillus licheniformis* DW2 has shown a multi-factor mechanism, including increasing the NADPH supply, changing the cell morphology and membrane composition, and remodeling metabolic pathways [23]. The PDR1 mutant of *S. cerevisiae* was screened by adaptive evolution, which resisted the damage of 2-PE to the cell membrane by increasing the ratio of unsaturated fatty acids/saturated fatty acid [24]. To more effectively produce 2-PE, the *GSH2* gene was screened based on a plasmid-based genomic library, which increased the 2-PE tolerance of *Candida glycerinogenes* and increased 2-PE production (16.2% higher) [25]. Therefore, the development of a robust strain is necessary for more efficient 2-PE production.

In this study, transcriptome analysis and metabolic engineering were used to improve the biosynthesis of 2-PE. By screening the key components of the amino acid transport module, aminotransferase module, decarboxylation module, dehydrogenase module, and 2-PE response module, hyper 2-PE producing strains were obtained. Subsequently, based on a transcriptomics assay, two novel target genes related to 2-PE resistance, *PDR12* and *C4R2I5*, were identified and successfully applied to improve the 2-PE resistance of the hyper 2-PE-producing strain. Here, we combined metabolic engineering with tolerance engineering strategies to identify key genes in the 2-PE synthesis pathway in *K. phaffii*, enriching the understanding of aromatic alcohol metabolic pathways and resistance mechanisms. The engineering strategy proposed in this paper is an example strategy to increase the production of 2-PE biosynthesis in yeast.

## Materials and methods

### Strains, media, and cultivation

*K. phaffii* GS115 was used as the host strain. The strains and plasmids used in the study are listed in Tables S2, S3 (Supporting Information). All yeast strains were cultivated in YPD media (10 g/L yeast extract, 20 g/L peptone, and 20 g/L glucose). The cells were incubated on a rotary shaker at 30 °C, and 200 rpm. The recombinant yeast strains were cultivated in MD medium (2% glucose, and 1.34% YNB without amino acids). *Escherichia coli* was grown in LB medium (10 g/L tryptone, 5 g/L yeast extract, and 10 g/L NaCl) at 37 °C. Ampicillin and kanamycin sulfate were added as needed. The fermentation medium contained 80 g/L glucose, 5 g/L l-phenylalanine, 6.7 g/L YNB without amino acids and ammonium sulfate, 0.5 g/L MgSO_4_, 5 g/L K_2_HPO_4_, 0.4 mg/L biotin, and 0.04 g/L histidine.

### Plasmid and recombinant strain construction

The gene was modified using CRISPR-Cas9 gene editing technology, and the strain was constructed using His4 as a selection marker. The pCas9 plasmid construction process was performed as previously reported [26]. The gRNA was selected through the chopchop website (https://chopchop.cbu.uib.no/). The detailed sequence of the gRNA is shown in Table S4. The insertion sites of the overexpressed genes were selected as previously reported [27]. The donor DNA was obtained by overlap PCR for gene fragment integration. A total of 1000 ng of donor DNA and 500 ng of CRISPR-Cas9 plasmids carrying gRNA were transferred into the cells by electroporation. The recombinant yeast strains were cultivated in MD medium at 30 °C for 5 days, after which several colonies were selected for PCR confirmation.

### Fermentation of 2-PE in a shake flask

The yeast cells were first inoculated into a tube containing 5 mL of medium and grown overnight at 30 °C with shaking at 250 rpm. Then, the cultures were inoculated into a 250 mL Erlenmeyer flask containing 50 mL of YPD medium and cultured in a shaking incubator under the same conditions for 24 h. The cultured broth was centrifuged to collect the cells, which were inoculated into a 250 mL Erlenmeyer flask containing 30 mL of fermentation medium. The initial OD_600_ was 6, and fermentation was completed after 48 h at 30 °C.

### Analytical methods

2-PE and l-Phe were analyzed and quantified using high-performance liquid chromatography (HPLC) system equipped with an Agilent Zorbax Eclipse plus C18 column (4.6 × 250 mm, 5 μm). The mobile phase was 50% methanol with a flow rate of 0.6 mL/min. The column temperature was 40 °C. The samples were centrifuged at 12,000×*g* for 10 min. Each supernatant was diluted with 5 volumes of ddH_2_O, and 10 μL of the diluted sample was injected. To detect the concentrations of residual glucose, HPLC was conducted using an Aminex HPX-87 H column (7.8 × 300 mm; Bio-Rad Laboratories, Inc, Hercules, CA, USA) at 55 °C with 5 mM sulfuric acid as the mobile phase. The injection volume and flow rate were set as 10 μL and 0.6 mL/min, respectively. The cell growth was monitored by OD_600_.

### RNA sequencing and data analysis

For RNA-seq experiments, strain GS115 was fermented in l-Phe fermentation medium and cultured for 48 h at 30 °C. Yeast cells in the fermentation broth were collected by cryogenic centrifugation at 4, 12, and 24 h and immediately frozen in liquid nitrogen. The strains cultivated to the mid-log phase were transferred to media supplemented with 1.5 g/L, 2 g/L, or 3 g/L 2-PE for 4 h with 10% inoculum. The cells were harvested, frozen immediately in liquid nitrogen, and stored at − 80 °C. Then, the samples were sent to Biomarker (Qingdao, China) for sequencing.

### RNA extraction and RT-qPCR

The strains cultivated to the mid-log phase were transferred to media supplemented with different concentrations of 2-PE for 4 h with 10% inoculum. The cells were then harvested and shock-frozen in liquid nitrogen for RNA extraction. Total RNA was prepared using a yeast RNAiso kit and reverse-transcribed into cDNA using a PrimeScript RT reagent kit.

RT-qPCR was conducted using TB Green® Premix EX Taq™ II (Tli RNaseH Plus; Takara Biotechnol, Dalian, China) and the StepOne Plus Real-Time PCR System. The 2^−ΔΔCt^ method was used for quantitative analysis of gene transcript levels.

### Evaluation of stress tolerance

The 2-PE tolerance of the strain was evaluated by semiquantitative dilution spot-plate and liquid culture assays. The semiquantitative dilution spot-plate assay was based on previous studies [28]. Each spot was tested three times independently, and the results are presented as the means of three independent measurements. The growth curves of the strains in YPD culture medium and YPD medium supplemented with 1.5 g/L 2-PE were measured with a spectrophotometer.

### Electron microscopy

Transmission electron microscopy (TEM) was used to observe morphological changes in the cells of the WT and ΔPDR12 strains. After the cells were washed, fixed, and sectioned in sequence [28], the samples were observed by TEM (HT7800, Hitachi, Ltd., Japan).

## Results and discussion

### Construction of a 2-phenylethanol-producing *K. phaffii* strain

*K. phaffii* is an excellent platform for producing exogenous proteins and has been used as a cell factory to produce high value-added chemicals in recent years [29, 30]. According to the genomic information, *K. phaffii* has all the elements of the 2-PE synthesis pathway, indicating that it may be a natural production strain. Fermentation experiments showed that 2-PE could be produced through the Ehrlich pathway and the de novo synthesis pathway. Approximately 60 mg/L 2-PE could be synthesized from glucose by strain GS115. The strain produced approximately 910 mg/L 2-PE through the Ehrlich pathway, illustrating that *K. phaffii* was capable of converting l-Phe to 2-PE. Our results showed that the production of 2-PE through the Ehrlich pathway is more effective than that through the de novo pathway in *K. phaffii* (Fig. 1B). Hence, the Ehrlich pathway was chosen as the target for metabolic engineering. Previous studies have shown that phenylpyruvate decarboxylase, which catalyzes the conversion of phenylpyruvate to phenylacetaldehyde, is a key rate-limiting enzyme [7, 31]. Two phenylpyruvate decarboxylases, KDC1 and KDC2, were found in *K. phaffii* via genome-wide analysis. First, *KDC1* and *KDC2* were knocked out. Compared with the control strain GS115, which produced 905 mg/L 2-PE in 72 h, the engineered strains ΔKDC1 and ΔKDC2 produced 750 mg/L and 613 mg/L 2-PE, respectively, in 72 h, exhibiting 16.67% and 31.9% lower 2-PE productivity (Fig. 1C). These results indicated that *KDC2* might play a major role in the Ehrlich pathway of *K. phaffii*. Notably, the deletion of *KDC2* significantly decreased the unit cell production capacity. This may be because cells use the nitrogen produced by l-Phe mainly for cell growth metabolism rather than for the production of 2-PE. Furthermore, the strain ΔKDC1ΔKDC2 could produce 383 mg/L 2-PE under the same fermentation conditions, implying that there were other elements that might be involved in this decarboxylation reaction.

The heterologous expression of *ARO10* in *K. phaffii* has been reported to produce 271 mg/L 2-PE [16]. Indeed, the overexpression of *KDC1* and *KDC2* also favored 2-PE synthesis. The introduction of the heterologous gene *KDC1* in *E. coli* resulted in a significant increase in 2-PE production to 57 mg/L [15]. Natural *Bacillus licheniformis* lacks phenylpyruvate decarboxylase and is unable to synthesize phenylacetaldehyde. By introducing *KDC2*, the pathway of synthesizing 2-PE from glucose was activated, which promoted the conversion of phenylpyruvate to phenylacetaldehyde, resulting in 30.42 mg/L 2-PE [32]. In our study, the overexpression of both *KDC1* (named S1) and *KDC2* (named S2) increased the production of 2-PE to 1.35 and 1.36 g/L, respectively. In addition, we compared the *ARO10* of the *S. cerevisiae* element, and no significant difference from the endogenous element of the overexpressed *K. phaffii* (Figure S1) was detected. Therefore, the endogenous genes of *K. phaffii* involved in the synthesis of 2-PE were identified via transcriptomics.

### Transcriptomic analysis of the effects of key genes in the Ehrlich pathway on the efficiency of 2-PE synthesis

To explore the potential candidate genes that could improve 2-PE biosynthesis in *K. phaffii*, we determined the dynamic changes in the expression of genes during batch culture by RNA-seq. In detail, the strain GS115 was sampled at 4 h, 12 h, and 24 h under l-Phe-supplemented fermentation conditions, and isochronous fermentation samples without l-Phe supplementation were used as controls. The transcriptome profiles showed that the transcription levels of 932 genes were significantly altered (|log2(fold change)| of > 1 and *P* value < 0.01) at 4 h, of which 620 were upregulated and 312 were downregulated. At 12 h, the transcription levels of 945 genes were significantly altered, of which 570 were upregulated and 3*75* were downregulated. The transcription levels of 941 genes were significantly altered at 24 h, of which 488 were upregulated and 453 were downregulated. Subsequently, the differentially expressed genes (DEGs) were classified using Kyoto Encyclopedia of Genes and Genomes (KEGG) annotations to assess their functions. The results suggested that most DEGs were associated with “nitrogen metabolism” and “carbohydrate metabolism” (Figure S2C), indicating that broad metabolic flux reprogramming was triggered by l-Phe supplementation. To identify the common DEGs among these three groups, the intersections between DEGs were also determined (Figure S2A). A total of 585 genes were significantly upregulated, and 500 genes were significantly downregulated during cultivation. In addition, RT-PCR was used to verify the reliability of the transcriptome data, which provided support for the in-depth identification of potential candidate genes for 2-PE synthesis (Figure S2B). According to the KEGG and GO analyses, a total of 33 DEGs involved in five modules were selected for further investigation (Table S5).

### Enhancement of 2-PE production via the Ehrlich pathway

**Fig. 2:**
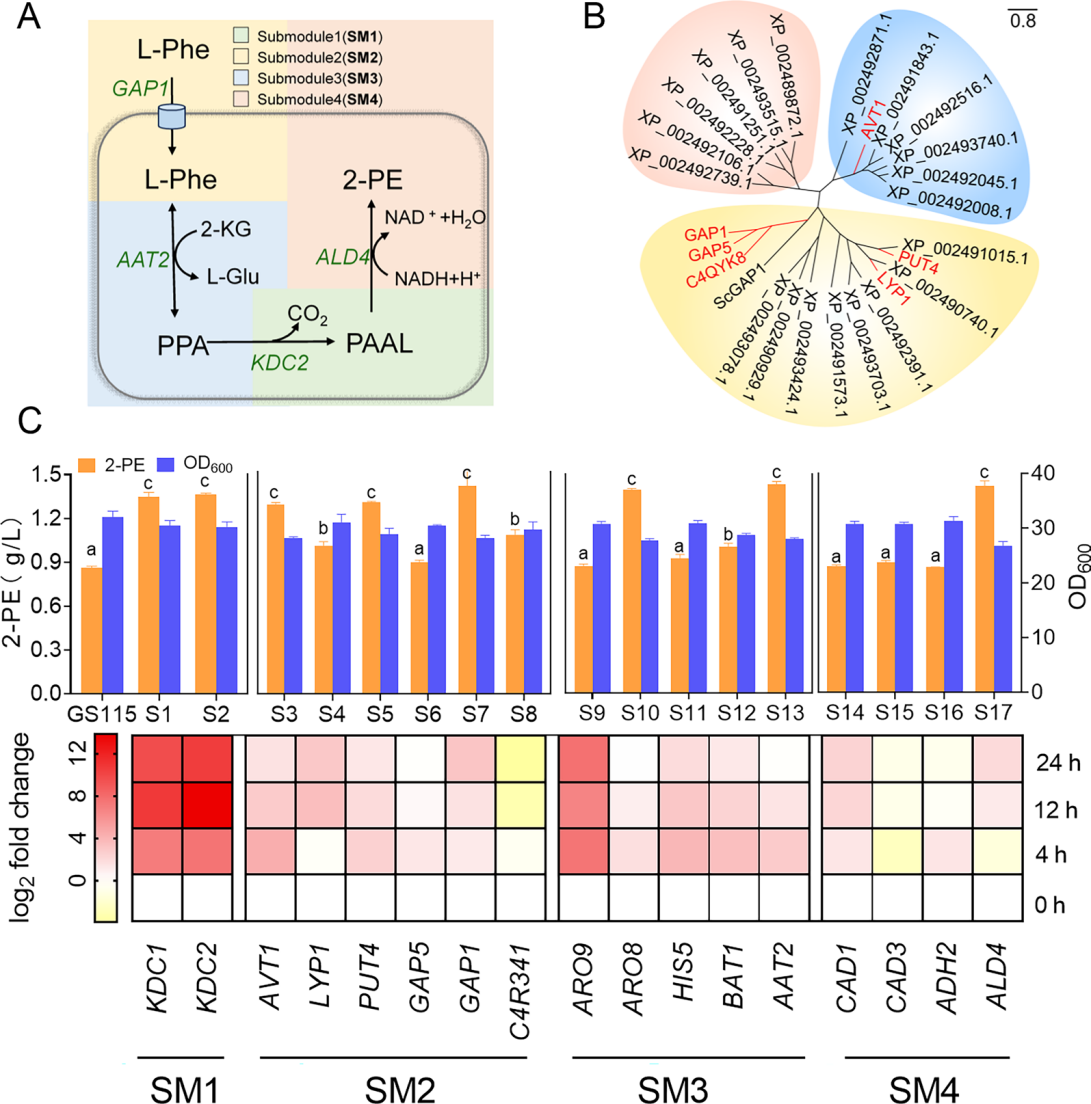
Analysis and validation of significantly upregulated and downregulated genes. **A** Modular schematic diagram of the Ehrlich pathway. **B** Evolutionary relationship of amino acid transporters in *K. phaffii*. **C** Changes in the transcription of SM1-4 DEGs at different time points and in the fermentation production and cell biomass of the recombinant strain 2-PE. The lowercase letters a, b, c, and d indicate significant differences as determined using Duncan’s multiple range test (*P* < 0.05)

According to the Ehrlich pathway, we divided these potential target genes into five modules based on functional annotation (Fig. 2A): the phenylpyruvate decarboxylation module (SM1), amino acid transport module (SM2), amino transfer module (SM3), and alcohol dehydrogenation module (SM4). To confirm the effect of these genes on 2-PE biosynthesis, the corresponding overexpression strains were constructed, and the ability of these recombinant strains to synthesize 2-PE was investigated (Fig. 2C). Yeast can use amino acids as nitrogen sources through various permeases [33, 34]. In this study, six permease genes whose transcription levels significantly changed were selected for further investigation. According to the phylogenetic analyses, except for AVT1, which is considered a vacuolar transporter, the other 5 amino acid permeases were concentrated in a cluster (Fig. 2B). These results suggested that l-Phe may be effectively absorbed by yeast cells through the participation of these genes, thereby increasing 2-PE production. Therefore, the expression of these permeases under the strong promoter *GCW14* is necessary to enhance substrate transport. Among these strains, strains overexpressing *AVT1* (S3), *PUT4* (S5), and *GAP1* (S7) exhibited outstanding performance in 2-PE synthesis. Strains S3, S5, and S7 significantly increased 2-PE production, producing 1.28, 1.3, and 1.4 g/L 2-PE, respectively (Fig. 2C). Compared with that of the S2 strain, the l-Phe consumption rate of the S7 strain (0.24 g/L/h) was the fastest in the first 8 h of fermentation. However, the transport rates of strains S3 and S5 were only 0.17 g/L/h (Figure S3). These results showed that *GAP1* and *PUT4*, which have a broad substrate spectrum, accelerate the transport of l-Phe from the extracellular to the intracellular space and increase the production of 2-PE. Surprisingly, the vacuolar transporter *AVT1* also enhanced the transport of l-Phe in cells, indicating that the regulation of intracellular amino acids also promoted the absorption of l-Phe in cells.

For the synthesis of 2-PE, improving the ability of aminotransferases to catalyze the synthesis of phenylpyruvate from l-Phe is very important. Previous studies have shown that increasing the expression of the aminotransferases ARO8 and ARO9 in *S. cerevisiae* could improve the conversion of l-Phe to phenylpyruvate [14, 35]. However, because of the broad substrate specificity of aromatic transaminases [34], the overexpression of transaminase might affect the metabolism of other amino acids. Therefore, according to transcriptome analysis, we selected five transaminase genes with upregulated gene expression levels for overexpression. *ARO8* (S10) and *AAT2* (S13) significantly increased the production of 2-PE to 1.4 g/L and 1.43 g/L, respectively (Fig. 2C). *BAT1* (S12) had a slight promoting effect on 2-PE synthesis. The expression of *ARO8* is regulated by a universal regulatory system for amino acid biosynthesis. *ARO8* plays the main catalytic role when the source of nitrogen in the medium is aromatic amino acids. Therefore, overexpression of *ARO8* enhances the transamination of l-Phe. *AAT2* encodes an aspartate aminotransferase that catalyzes the reversible transfer of an amino group from l-aspartate to 2-oxoglutarate to form oxaloacetate and l-glutamate [36]. Therefore, the broad substrate scope of this enzyme is believed to allow it to specifically transfer an amino group of l-Phe to α-ketoglutaric acid to produce l-glutamic acid and phenylpyruvate, thereby promoting the production of 2-PE.

The final step of the Ehrlich pathway is the generation of alcohols from aldehydes catalyzed by alcohol dehydrogenases, which transfer C4-hydride from NAD(P)H to the carbonyl carbon of the aldehyde substrate [14, 37]. Therefore, recombinant strains were constructed to verify the effect of differentially expressed alcohol dehydrogenase genes at different transcription levels in the transcriptome on 2-PE biosynthesis (Fig. 2C). The fermentation results showed that the 2-PE production of the *ADH2, CAD1,* and *CAD3* overexpression strains did not change significantly compared with that of the control strains, but the 2-PE production of the *ALD4-*overexpressing (S17) strain increased to 1.42 g/L. Characterization of the overexpression mutants indicated that *ALD4* is the main isoenzyme that catalyzes 2-PE formation. Previous studies have shown that *ALD4* mainly functions as an “acetaldehyde pump” in *S. cerevisiae* and can directly increase the flow rate of intracellular NADH, which may be the reason for the increase in 2-PE production [38].

**Fig. 3:**
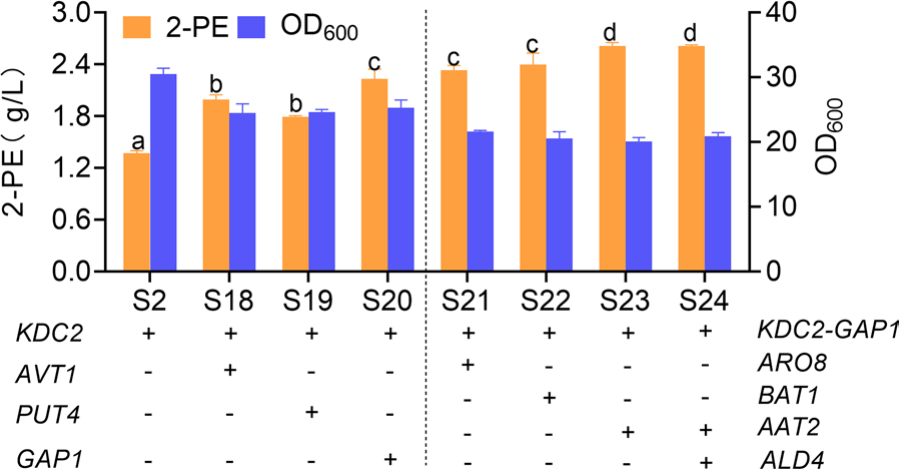
Combinatorial expression of Ehrlich pathway-related genes. The lowercase letters a, b, c, and d indicate significant differences as determined using Duncan’s multiple range test (*P* < 0.05)

Previous studies have reported that the use of promoter replacement engineering to increase the expression of genes related to the Ehrlich pathway increased 2-PE production by 37% [11]. In the above experiments, we found that the amino acid permeases, transaminases, and alcohol dehydrogenase genes *AVT1, PUT4, GAP1, ARO8, AAT2,* and *ALD4* were suitable for the production of 2-PE in *K. phaffii*. Here, the potential genes from the four modules were expressed in combination, and the most suitable gene combinations for 2-PE production were explored. Although the production of 2-PE increased due to the overexpression of phenylalanine decarboxylase, l-Phe remained in the fermentation broth at the end of fermentation, which may be due to insufficient absorption of l-Phe by the microorganisms. Therefore, the amino acid permeases *AVT1, PUT4,* and *GAP1* were overexpressed in strain S2, and the corresponding strains S18, S19, and S20 were generated to enhance the transport of l-Phe (Fig. 3). The addition of amino acid permease further increased the production of 2-PE, and the productions of S18, S19, and S20 were 1.99, 1.85, and 2.24 g/L, respectively. However, because phenylalanine remained in the medium, we believe that other rate-limiting steps that affect the metabolism of phenylalanine are present in the pathway. Therefore, the next step was to increase the intracellular utilization of l-Phe. To promote the transfer of amino acids to produce phenylpyruvate, the transaminases *ARO8, BAT1* and *AAT2* were overexpressed to produce the corresponding strains S21, S22, and S23. As expected, the production of 2-PE increased further. The 2-PE production of strain S23 was the highest, reaching 2.61 g/L. *ALD4* (S24) was subsequently overexpressed on the basis of strain S21 to increase the ability to synthesize 2-phenylacetaldehyde from 2-PE. Unfortunately, the introduction of *ALD4* did not increase 2-PE production (Fig. 3).

In brief, stepwise refactoring of the Ehrlich pathway led us to identify the optimal catalytic modules consisting of l-Phe permease, aromatic amino acid aminotransferase, phenylpyruvate decarboxylase, and alcohol dehydrogenase.

### Combining modular engineering with global gene regulation to improve the efficiency of 2-PE synthesis

**Fig. 4:**
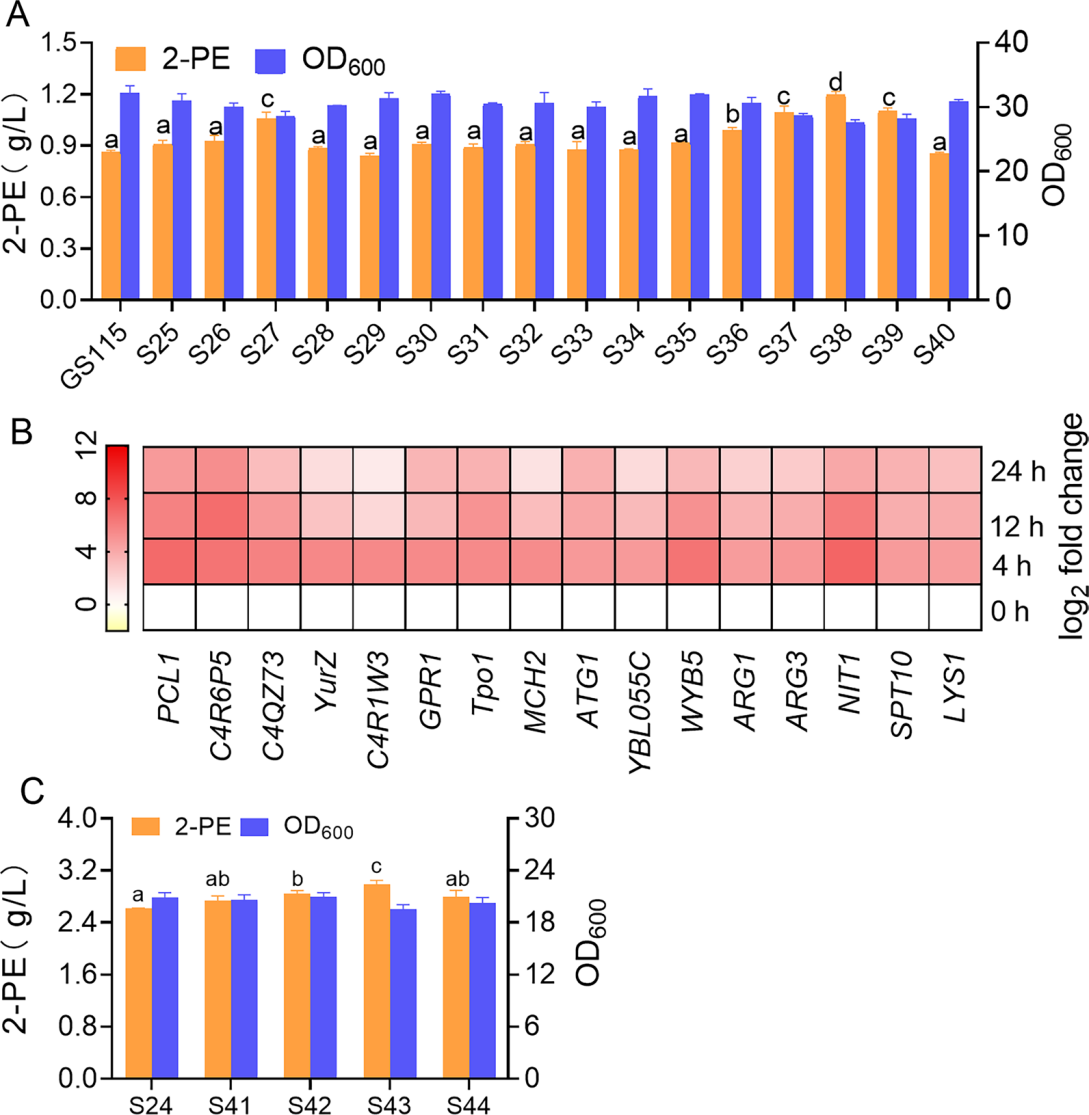
Analysis of significantly up-regulated genes. **A** Validation of the effects of significantly differentially expressed genes on 2-PE biosynthesis. **B** The transcriptional changes of differentially expressed genes at different time points. **C** Effects of transcriptome up-regulated genes in modular engineered strain S24 on 2-PE synthesis. The values are presented as the means ± SDs (*n* = 3). The lowercase letters a, b, c and d indicate significant differences according to Duncan’s multiple range test (*P* < 0.05)

The above results indicated that Ehrlich pathway-related genes promoted the synthesis of 2-PE. The interaction between the synthetic pathway and the entire metabolic network of the chassis cells also significantly affects the synthesis efficiency of the products. To determine the effect of l-Phe as a nitrogen source for the synthesis of 2-PE on the global metabolic network of *K. phaffii*, based on transcriptome analysis, we used the strong promoter *TEF1* to control 16 genes whose transcription levels were significantly upregulated (SM5, Fig. 4B). The fermentation results showed that overexpression of *C4QZ73, ARG3, NIT1,* and *SPTI10* could increase the 2-PE titer by more than 20% compared with that of the control strain GS115, and the corresponding recombinant strains were S27, S37, S38, and S39 (Fig. 4A). Among them, strain S38 produced the highest 2-PE titer, reaching 1.19 ± 0.12 g/L.

To assess the effect of these four genes on 2-PE production, we used S24 as a production host to further overexpress the genes *C4QZ73, ARG3, NIT1,* and *SPTI10* under the control of the *TEF1* promoter to produce strains S41–S44. The fermentation results showed that the 2-PE titer of strains S41–S44 was mostly greater than that of S24, and the 2-PE production of strain S43 was the highest (2.98 ± 0.02 g/L), which was 14.2% greater than that of S24 (Fig. 4C). At this time, the final biomass (OD_600_) of the shake flask fermentation system was 20, and the biomass decreased by 38% compared with that of the GS115 fermentation system. Since the current 2-PE production reached 2.98 g/L, we believe that the toxicity of the high-concentration products inhibited cell growth. These environmental stresses are among the important bottlenecks that limit the use of microbial cells for industrial production. By modifying the antistress element stp15 gene of *S. cerevisiae* via genetic engineering, the tolerance and production of the engineered strain were significantly improved. Therefore, increasing the cell tolerance to 2-PE might increase the production of 2-PE [39].

### Mutant characterization to determine the contribution of specific genes to 2-PE tolerance

**Fig. 5:**
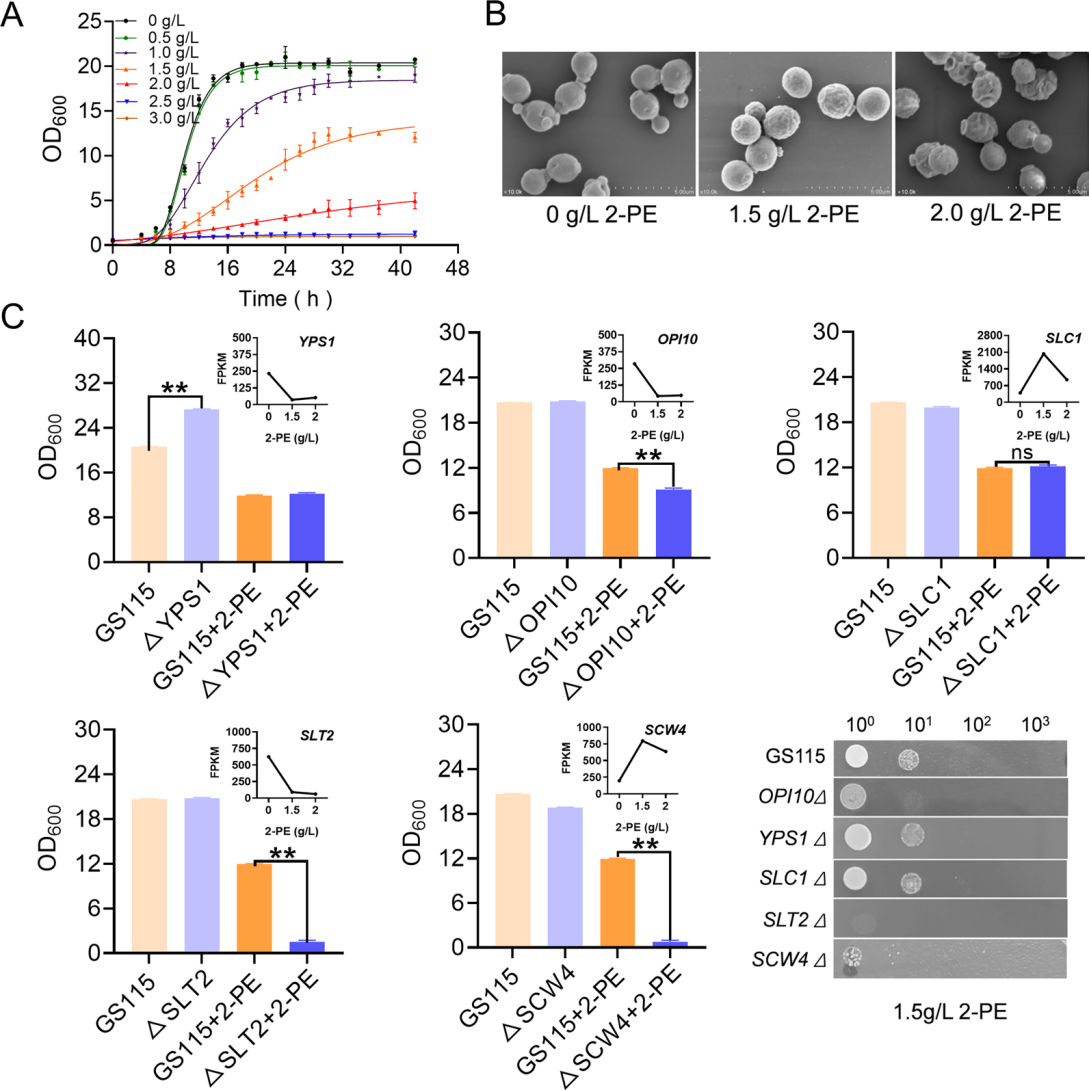
Cell growth of the strain under 2-PE stress. **A** Growth curves of GS115 under different concentrations of 2-PE. **B** The cell morphology of strain GS115 under different concentrations of 2-PE. **C** Transcription levels of differentially expressed genes at different 2-PE concentrations and biomasses of differential gene deletion strains with or without 1.5 g/L 2-PE. The values are presented as the means ± SDs (*n* = 3). Statistical significance is denoted as **P < 0.01

Cytotoxicity toward host cells limits microbial growth and prevents efficient microbial production of aromatic compounds. To investigate the 2-PE tolerance of GS115, the growth capacity of GS115 was measured under different concentrations of 2-PE stress. As shown in Fig. 5A, the growth curve revealed that the growth inhibition of 2-PE in GS115 increased significantly with increasing concentrations of 2-PE. Although the cells could still grow under 2 g/L 2-PE stress, the biomass was 23.86% of that under nonstress conditions. When the concentration of 2-PE reached 2.5 g/L, cell growth was completely inhibited. Changes in the cell morphology of the strain under 2-PE stress were observed by scanning electron microscopy. After 2-PE stress, the surface of the cell membrane became rough, wrinkled, atrophied, and fractured (Fig. 5B). These results indicated that the toxicity of 2-PE led to a decrease in cell viability, which may inhibit the production of GS115. Alleviating 2-PE toxicity or increasing host strain 2-PE tolerance has been reported to improve 2-PE productivity [40, 41]. In the face of the complexity and global nature of the microbial cell response to stress, researchers have mainly used omics techniques to explain microbial tolerance [42, 43]. To do this, we conducted high-throughput sequencing of the wild-type strain GS115 exposed to a gradient of 2-PE (0, 1.5, and 2.0 g/L) for 4 h. Transcription analysis revealed that the expression levels of 787 genes were significantly changed (FDR ≤ 0.05, FC ≥ 2) under two different concentrations of 2-PE, of which 466 were upregulated and 321 were downregulated (Figure S4). According to KEGG analysis, these DEGs were mainly involved in oxidative phosphorylation, fatty acid synthesis and metabolism, glutathione metabolism and synthesis, and degradation of ketone bodies (Figure S5).

Further analysis revealed that the DEGs were primarily associated with glutathione metabolism, endoplasmic reticulum processing and synthesis, and ABC transport. To investigate the contribution of these genes to 2-PE tolerance, knockout mutants were constructed (Fig. 5C). Previous research has indicated that the cell membrane is the primary target of 2-PE toxicity. When exposed to 2-PE stress, the integrity of the cell membrane was damaged. Phospholipids are the main structure of biofilms and determine their fluidity and flexibility. Therefore, the *OPI10*, *YPS1,* and *SLC1* DEGs related to phospholipid synthesis were knocked out. The tolerance of the *OPI10* knockout strain to 2-PE decreased, which may have been caused by increased cell permeability and decreased cell activity. The deletion of *YPS1* did not change the tolerance of cells to 2-PE. Consistent with the previous reports, *YPS1* knockout increased cell biomass without causing stress [44]. The transcription level of the *SLC1* gene in *Candida glycerinogenes* increased 73-fold under 2-PE stress [45]. Overexpression of the *SLC1* gene increased the tolerance of the strain to 2-PE, and the biomass increased by 18.3%. Meanwhile, knockout of the *SLC1* gene in *K. phaffii* did not affect the tolerance of cells to 2-PE in this study. This result suggested that the function of *SLC1* was not highly conserved among closely related yeast species, which limits its application. In addition, when the cell membrane integrity genes *SLT2* and *SCW4* were knocked out, the mutants almost completely lost their tolerance to 2-PE, which may be due to the destruction of cell membrane integrity and leakage of intracellular substances. Consequently, the integrity of the cell membrane is crucial for the resistance of the strain to damage by exogenous 2-PE.

### The *PDR12* gene enhanced strain tolerance to 2-PE

**Fig. 6:**
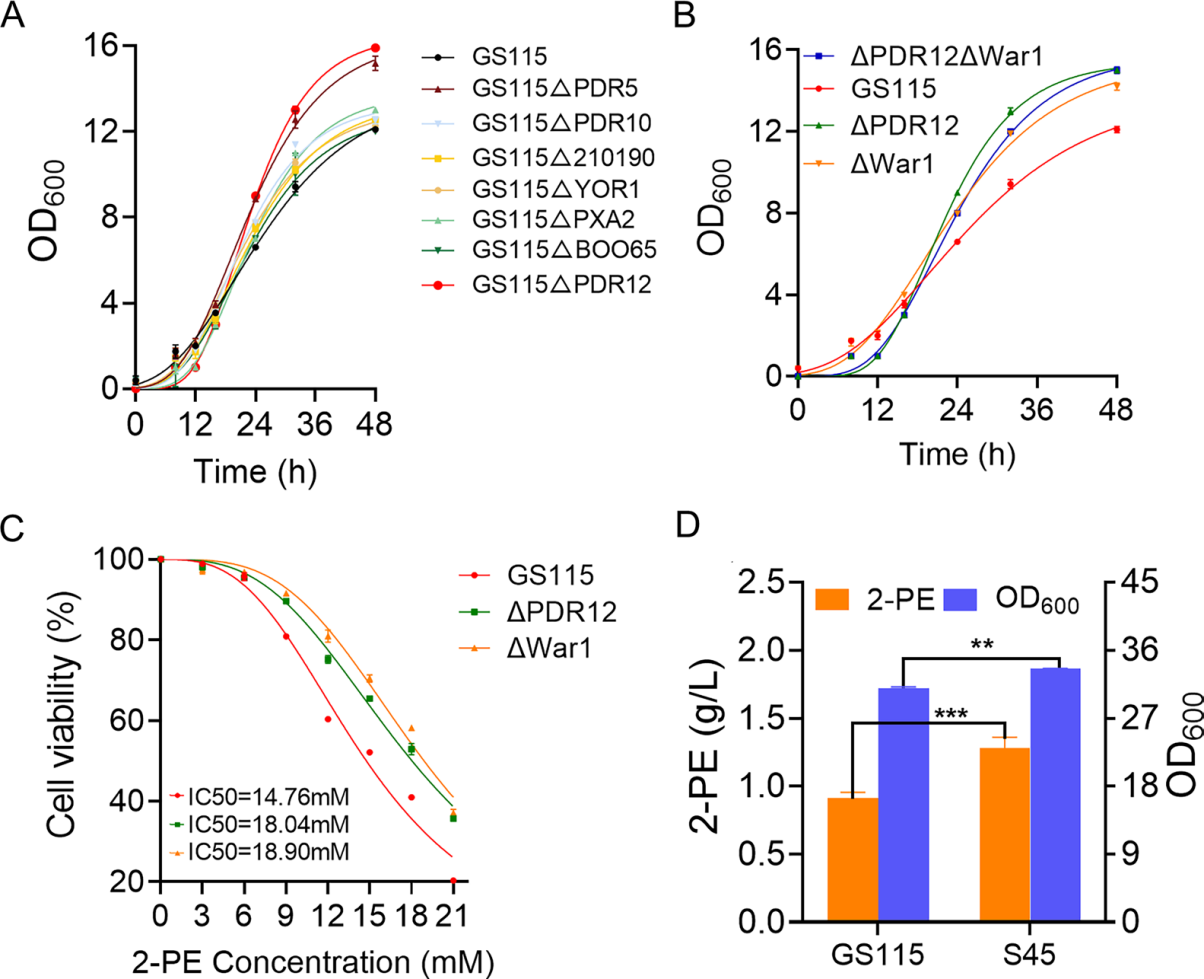
Screening and validation tests for 2-PE tolerance genes in *K. phaffii*. **A** The growth curve of the differential gene knockout strain under 1.5 g/L 2-PE stress. The growth curve and IC50 of (**B**–**C**) the wild-type strain (GS115), the ΔPDR12 strain, the ΔWar1 strain, and the ΔPDR12ΔWar1 double knockout strain under 1.5 g/L 2-PE stress. **D** 2-PE produced by the fermentation of strain ΔPDR12. The values are presented as the means ± SDs (*n* = 3). Statistical significance is denoted as ***P < 0.001, **P < 0.01

**Fig. 7:**
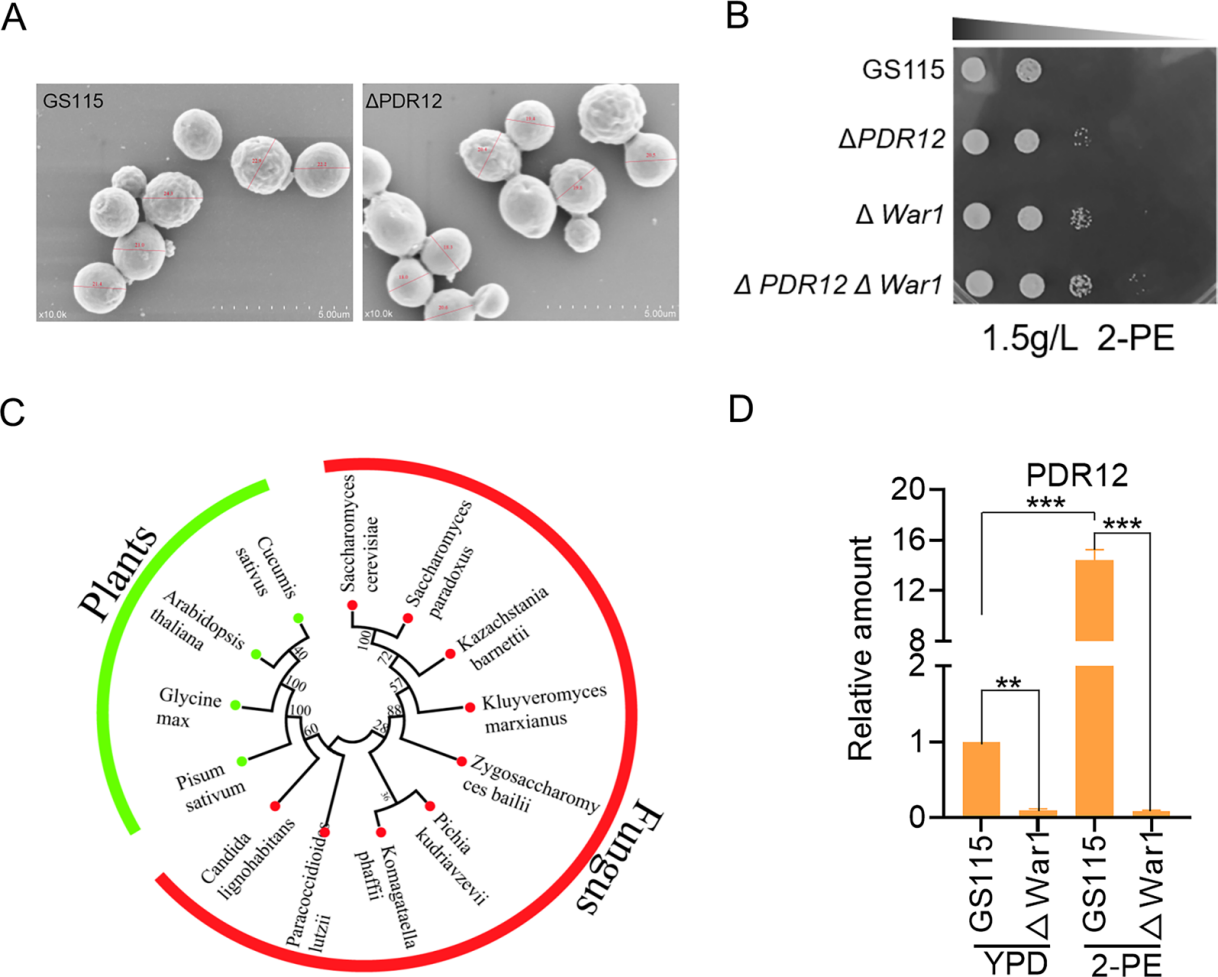
Changes in the ΔPDR12 strain under 2-PE stress. **A** The cell morphology of the ΔPDR12 strain under 2-PE stress. **B** Growth of the recombinant strain on the dilution spot-plate assay under 2-PE stress. **C** PDR12 phylogenetic tree among different strains. **D** The transcription level of *PDR12* in the ΔWar1 strain under 2-PE stress was altered. The values are presented as the means ± SDs (*n* = 3). Statistical significance is denoted as ***P < 0.001, **P < 0.01

DEGs were also enriched in ABC transporters, and their transcriptional levels are shown in Table S6. ABC transporters are related to resistance to various chemical stresses and are involved in the detoxification of these compounds. These genes are potential target genes for improving 2-PE tolerance. As shown in Fig. 6A, ΔPDR5 exhibited reduced sensitivity to 2-PE, and its biomass increased by 28.9%. The strain ΔPDR12 showed the best tolerance under 1.5 g/L 2-PE stress. Compared with GS115, the IC50 value of the ΔPDR12 strain reached 18.04 mM, increased by 22.22%, and the biomass increased by 34.1% (Fig. 6B and C). Electron microscopy revealed that the cell size of the ΔPDR12 strain decreased and the specific surface area increased under 2-PE stress (Fig. 7A). Strain ΔPDR12 may alleviate 2-PE inhibition by accelerating the exchange of nutrients between cells and the external environment. In this study, strain ΔPDR12 not only improved the tolerance to aromatic alcohols, but also improved the 2-PE synthesis ability of the strain. The production of 2-PE in the ΔPDR12 strain was 1.28 g/L. Compared with those of GS115, the final biomass and production increased by 8.1% and 40.6%, respectively (Fig. 6D). A phylogenetic analysis demonstrated that *PDR12* is widely distributed in other eukaryotic species (Fig. 7C). This study analyzed the evolutionary relationship of *PDR12* among different species and revealed that *PDR12* is a conserved gene that widely exists in fungi. The phylogenetic tree showed that *K. phaffii PDR12* was closely related to *Pichia kudriavzevii PDR12*. Compared with the distance between *K. phaffii* and *S. cerevisiae* on the evolutionary tree, *K. phaffii* is closer to other nonconventional yeasts. Therefore, the *PDR12* gene might have conserved functions in 2-PE resistance in these species.

The induction of *PDR12* under sorbitol stress is regulated by the transcription factor War1. To explore the relationship between *PDR12* and 2-PE, War1 was first knocked out on the basis of GS115. The tolerance of strain ΔWar1 to 2-PE was consistent with that of strain ΔPDR12. The results of the dilution spot-plate and IC50 assays showed that the knockout of the upstream regulatory factor War1 increased the tolerance of cells to 2-PE (Fig. 7B). The *PDR12* transcription levels of GS115 and the ΔWar1 strain with or without 2-PE stress were also detected (Fig. 7D). Under 2-PE stress, the transcription level of the *PDR12* gene in the GS115 strain increased significantly, indicating that 2-PE activated the expression of the *PDR12* gene in response to changes in the external environment, which was consistent with our transcriptome data. However, the expression level of the *PDR12* gene in the ΔWar1 strain was significantly downregulated with or without 2-PE stress, and the transcription level was very low. These results indicated that 2-PE activated the transcription factor War1 to regulate the expression of *PDR12*. To further investigate the relationship between *War1* and *PDR12*, the double knockout strain ΔPDR12ΔWar1 was constructed. The growth profiles and tolerance assessment showed that the ΔPDR12, ΔWar1, and ΔPDR12ΔWar1 strains exhibited the same tolerance to 2-PE, and they all showed a significant increase compared with that of GS115 (Fig. 6B). This finding also indicates that PDR12 is regulated by War1 under 2-PE stress. The increased tolerance to 2-PE caused by War1 knockout was also caused by the low expression of *PDR12*, suggesting that these genes are involved in the same signaling pathway.

### Transcriptome analysis of the PDR12 mutant

**Fig. 8:**
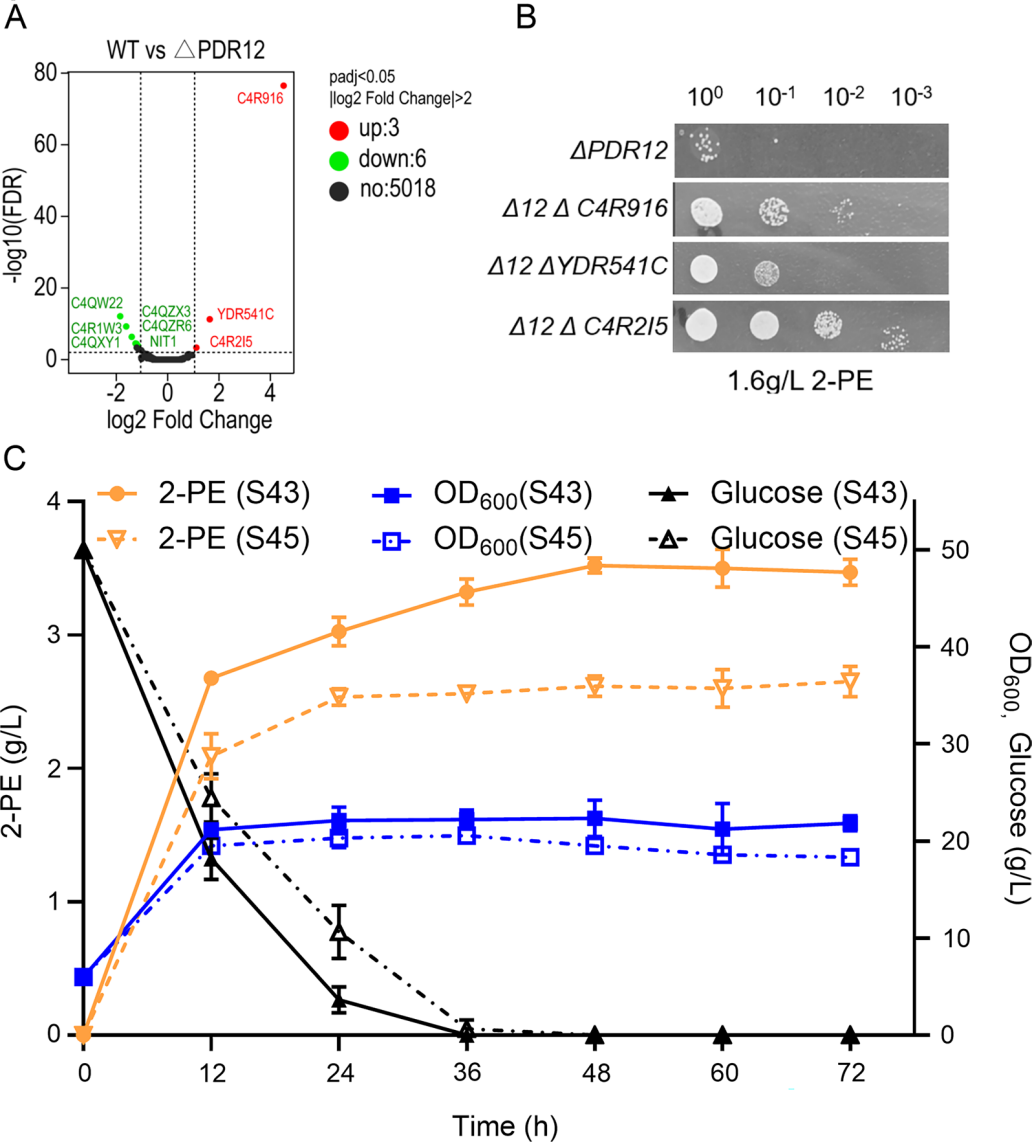
The elimination of *C4R2I5* improved the tolerance and production of 2-PE in yeast. **A** Differentially expressed genes in the transcriptome of the GS115 and PDR12 strains were knocked out under 2-PE conditions. **B** The dilution spot-plate assay showed that differential gene knockout improved *K. phaffii* tolerance to 2-PE. **C** Flask fermentation experiment of engineered strains

*PDR12* has previously been shown to be associated with the plasma membrane and stress tolerance. To further study the relationship between *PDR12* and 2-PE, we determined the changes in the expression of genes during batch culture of ΔPDR12 via RNA-seq. There were only 9 DEGs, of which 3 genes were significantly upregulated and 6 genes were significantly downregulated (Fig. 8A). Some DEGs were involved in cell respiration (*C4QZX3*), coenzyme transport metabolism (*C4QXY1*), and amino acid transport and metabolism (*NIT1*). However, four of the nine differentially expressed genes (*C4R1W3*, *C4QZX3*, *C4QZR6*, and *C4R2I5*) are unique and only exist in *K. phaffii*. To assess the contributions of these genes, double mutants were generated, and their tolerances to 2-PE were determined. Compared with that of the ΔPDR12 strain, the tolerance of the C4R2I5 knockout strain to 2-PE significantly increased. Knockout of the *C4R916* and *YDR541C* genes was also beneficial for enhancing the tolerance of the strain to 2-PE, but the effect was lower than that of *C4R2I5* gene knockout (Fig. 8B). The *C4R2I5* gene is expressed only in *K. phaffii*, and its function has not been reported. At present, the *C4R916* and *YDR541C* genes have been confirmed to have detoxification effects only on furfural. The *C4R916* and *YDR541C* genes are predicted to encode aldehyde reductases, and aromatic aldehydes can be used as substrates for these enzymes. The deletion of the *C4R916* and *YDR541C* genes improved the tolerance of the strain to 2-PE. Reducing the conversion of aromatic aldehydes to aromatic alcohols may reduce the stress of the toxic product 2-PE on the strain. In short, the identification of these novel components helps to further define the complex mechanisms involved in 2-PE tolerance.

### Improvement of 2-PE production using tolerance engineering

Currently, tolerance engineering is a promising method for enhancing the robustness of cell factories [46]. In our above experiment, the *PDR12* and *C4R2I5* knockout strains exhibited reduced cell sensitivity. To further improve 2-PE production, *PDR12* and *C4R2I5* were knocked out in strain S43, generating strain S45. Compared with strain S43, strain S45 showed much faster growth and glucose consumption (Fig. 8C). Strain S45 produced a 2-PE production of 3.02 g/L within 24 h, and its 2-PE production increased by 11.4% compared with that of the control strain S43. In addition, the 2-PE production of strain S45 was 3.52 g/L, and the conversion rate was 0.704 g/g l-Phe. This is currently the highest production of 2-PE produced by *K. phaffii*. These results indicated that tolerance engineering can improve the tolerance of the strain, alleviate the inhibitory effects of toxic substances on cell growth, and effectively enhance the production performance of the strain.

## Conclusions

In this work, genes that increase 2-PE production were obtained through transcriptome analysis and phenotypic validation, and modular expression was performed. When the genes related to the 2-PE anabolic network were further optimized, the production was not further improved, which may be due to the serious toxicity of the product to cells, hindering the effective production of 2-PE. Therefore, the genes related to the tolerance of *K. phaffii* to 2-PE were analyzed based on the transcriptome. Knockout of the *PDR12* and *C4R2I5* genes enhanced the tolerance of yeast to 2-PE, and *PDR12* responded to 2-PE stress through the upstream transcription factor War1. Moreover, knockout of *PDR12* and *C4R2I5* also increased the production of 2-PE. Through metabolic engineering and tolerance engineering, the ability of *K. phaffii* to synthesize 2-PE was enhanced, showing its potential for 2-PE production. In addition, our study revealed the role of *PDR12* in 2-PE tolerance. These results provide valuable insights into the future production of aromatic compounds in *K. phaffii* and the development of efficient and robust yeast strains.

### Supplementary Information

Supplementary Material 1. Table S1: Summary of 2-PE production by microorganisms. Table S2: Strains used in this study. Table S3: Plasmids used in this study. Table S4: gRNA used in this study. Table S5: 33 differential target genes identified in this study. Table S6: Differentially expressed ABC transporter genes at the transcriptional level. Figure S1: Fermentation production of 2-PE obtained by overexpressing the phenylalanine decarboxylase strain of *S. cerevisiae*. Figure S2: Transcriptome analysis of strains without L-Phe feeding and with L-Phe feeding for 4, 12 and 24 h. Figure S3: The consumption of L-Phe in the transporter-overexpressing strain at 8 h and 16 h. Figure S4: Volcano plot of differentially expressed genes in the strains after 4 h of 0 g/L 2-PE stress and 4 h of 1.5 g/L 2-PE and 2 g/L 2-PE stress. Figure S5: KEGG enrichment bubble diagram of the DEGs in the strains after 4 h of 0 g/L 2-PE, 1.5 g/L 2-PE or 2 g/L 2-PE stress. Figure S6: Growth curve of the recombinant strain under 1.5 g/L 2-PE stress.

## Data Availability

The data collected upon which this article is based upon are all included in this manuscript and the additional files associated with it.
