# Supplementary material for: Metabolic and tolerance engineering of Komagataella phaffii for 2-phenylethanol production through genome-wide scanning
Source: Biotechnol Biofuels Bioprod. 2024 Jul 22;17:107. doi: 10.1186/s13068-024-02536-y (PMC11265028; doi:10.1186/s13068-024-02536-y)
Supplement: Supplementary file 1 — Supplementary Material 1. Table S1: Summary of 2-PE production by microorganisms. Table S2: Strains used in this study. Table S3: Plasmids used in this study. Table S4: gRNA used in this study. Table S5: 33 differential target genes identified in this study. Table S6: Differentially expressed ABC transporter genes at the transcriptional level. Figure S1: Fermentation production of 2-PE obtained by overexpressing the phenylalanine decarboxylase strain of S. cerevisiae. Figure S2: Transcriptome analysis of strains without L-Phe feeding and with L-Phe feeding for 4, 12 and 24 h. Figure S3: The consumption of L-Phe in the transporter-overexpressing strain at 8 h and 16 h. Figure S4: Volcano plot of differentially expressed genes in the strains after 4 h of 0 g/L 2-PE stress and 4 h of 1.5 g/L 2-PE and 2 g/L 2-PE stress. Figure S5: KEGG enrichment bubble diagram of the DEGs in the strains after 4 h of 0 g/L 2-PE, 1.5 g/L 2-PE or 2 g/L 2-PE stress. Figure S6: Growth curve of the recombinant strain under 1.5 g/L 2-PE stress. [file 13068_2024_2536_MOESM1_ESM.docx]

**Supporting Information**

Metabolic and tolerance engineering of *Komagataella phaffii* for 2-phenylethanol production through genome-wide scanning

Lijing Sun, Ying Gao, Renjie Sun, Ling Liu, Liangcai Lin^*^, Cuiying Zhang^*^

State Key Laboratory of Food Nutrition and Safety, Key Laboratory of Industrial Fermentation Microbiology, Ministry of Education, Tianjin Key Laboratory of Industrial Microbiology, College of Biotechnology, Tianjin University of Science and Technology, Tianjin, 300457, People's Republic of China

Corresponding Author

^*^Email: lclin@tust.edu.cn; cyzhangcy@tust.edu.cn

**Supplementary** **Tables**

**Table S1**: Summary of 2-PE production by microorganisms.

| Strain | pathway | Cultivation | Production (g/L) | Reference |
| --- | --- | --- | --- | --- |
| *Pichia pastoris* | de novo | Shake flask | 1.17 g/L | ^1^ |
| *Saccharomyces cerevisiae* | de novo | 2 L Bioreactor | 1.59 g/L | ^2^ |
| *S. cerevisiae*  BY4741 | de novo | Shake flask | 0.41 g/L | ^3^ |
| *Yarrowia lipolytica* | de novo | Shake flask | 2.43 g/L | ^4^ |
| *Kluyveromyces marxianus* | de novo | Shake flask | 0.85 g/L | ^5^ |
| *Kluyveromyces marxianus* | de novo | Shake flask | 1.94 g/L | ^6^ |
| *E. coli* DG02 | de novo | Shake flask | 1.02 g/L | ^7^ |
| *Bacillus licheniformis* | de novo | Shake flask | 6.24 g/L | ^8^ |
| *Yarrowia lipolytica* | Ehrlich | Shake flask  (8 g/L L-Phe) | 2.67 g/L | ^9^ |
| *S. cerevisiae* YS58 | Ehrlich | 5 L Bioreactor  (9 g/L L-Phe) | 6.3 g/L | ^10^ |
| *Saccharomyces bayanus* | Ehrlich | 5 L Bioreactor  (12 g/L L-Phe) | 6.5 g/L | ^11^ |
| *S. cerevisiae* YS58 | Ehrlich | Shake flask  (5.5 g/L L-Phe) | 3.73 g/L | ^12^ |
| *S. cerevisiae* T2 | Ehrlich | Shake flask  (5 g/L L-Phe) | 3.59 g/L | ^13^ |
| *S. cerevisiae* S288c | Ehrlich | 2.5 L Bioreactor  (7 g/L L-Phe) | 2.61 g/L | ^14^ |
| *Candida glycerinogenes* | Ehrlich | Shake flask  (7 g/L L-Phe) | 5.0 g/L | ^15^ |
| *Bacillus licheniformis* | Ehrlich | Shake flask  (5 g/L L-Phe) | 3.04 g/L | ^16^ |

**Table S2.** Strains used in this study.

| Names | Characteristics | |
| --- | --- | --- |
| Strains |  |  |
| GS115 | his4- |  |
| S1 | GS115 derivate, ∷*P_TEF_-KDC1-T_PGK_* | This study |
| S2 | GS115 derivate, ∷*P_TEF_-KDC2-T_PGK_* | This study |
| S3 | GS115 derivate, ∷*P_GCW14_-AVT1-T_ALD2_* | This study |
| S4 | GS115 derivate, ∷*P_GCW14_-LYP1-T_ALD_****_2_*** | This study |
| S5 | GS115 derivate, ∷*P_GCW14_-PUT4-T_ALD2_* | This study |
| S6 | GS115 derivate, ∷*P_GCW14_-GAP5-T_ALD2_* | This study |
| S7 | GS115 derivate, ∷*P_GCW14_-GAP1-T_ALD2_* | This study |
| S8 | GS115 derivate, ∷*P_GCW14_-C4QYK8-T_ALD2_* | This study |
| S9 | GS115 derivate, ∷*P_GAP_-ARO9-T_AOX1_* | This study |
| S10 | GS115 derivate, ∷*P_GAP_-ARO8-T_AOX1_* | This study |
| S11 | GS115 derivate, ∷*P_GAP_-HIS5-T_AOX1_* | This study |
| S12 | GS115 derivate, ∷*P_GAP_-BAT1-T_AOX1_* | This study |
| S13 | GS115 derivate, ∷*P_GAP_-AAT2-T_AOX1_* | This study |
| S14 | GS115 derivate, ∷*P_PGK_-CAD1-T_GAP_* | This study |
| S15 | GS115 derivate, ∷*P_PGK_-CAD3-T_GAP_* | This study |
| S16 | GS115 derivate, ∷*P_PGK_-ADH2-T_GAP_* | This study |
| S17 | GS115 derivate, ∷*P_PGK_-ALD4-T_GAP_* | This study |
| S18 | S2 derivate, ∷*P_GCW14_-AVT1-T_ALD2_* | This study |
| S19 | S2 derivate, ∷*P_GCW14_-PUT4-T_ALD2_* | This study |
| S20 | S2 derivate, ∷*P_GCW14_-GAP1-T_ALD2_* | This study |
| S21 | S20 derivate, ∷*P_GAP_-ARO8-T_AOX1_* | This study |
| S22 | S20 derivate, ∷*P_GAP_-BAT1-T_AOX1_* | This study |
| S23 | S20 derivate, ∷*P_GAP_-AAT2-T_AOX1_* | This study |
| S24 | S23derivate, ∷*P_PGK_-ALD4-T_GAP_* | This study |
| S25 | GS115 derivate, ∷*P_TEF_-PCL1-T_PET9_* | This study |
| S26 | GS115 derivate, ∷*P_TEF_-* *C4R6P5-T_PET9_* | This study |
| S27 | GS115 derivate, ∷*P_TEF_-* *C4QZ73-T_PET9_* | This study |
| S28 | GS115 derivate, ∷*P_TEF_-* *YurZ-T_PET9_* | This study |
| S29 | GS115 derivate, ∷*P_TEF_-* *C4R1W3-T_PET9_* | This study |
| S30 | GS115 derivate, ∷*P_TEF_-* *GPR1-T_PET9_* | This study |
| S31 | GS115 derivate, ∷*P_TEF_-* *Tpo1-T_PET9_* | This study |
| S32 | GS115 derivate, ∷*P_TEF_-* *MCH2-T_PET9_* | This study |
| S33 | GS115 derivate, ∷*P_TEF_-* *ATG1-T_PET9_* | This study |
| S34 | GS115 derivate, ∷*P_TEF_-* *YBL055C-T_PET9_* | This study |
| S35 | GS115 derivate, ∷*P_TEF_-* *WYB5-T_PET9_* | This study |
| S36 | GS115 derivate, ∷*P_TEF_-* *ARG1-T_PET9_* | This study |
| S37 | GS115 derivate, ∷*P_TEF_-* *ARG3-T_PET9_* | This study |
| S38 | GS115 derivate, ∷*P_TEF_-NIT1-T_PET9_* | This study |
| S39 | GS115 derivate, ∷*P_TEF_-SPT10-T_PET9_* | This study |
| S40 | GS115 derivate, ∷*P_TEF_-LYS1-T_PET9_* | This study |
| S41 | S24 derivate, ∷ *P_TEF_-C4QZ73-T_PET9_* | This study |
| S42 | S24 derivate, ∷ *P_TEF_-ARG3-T_PET9_* | This study |
| S43 | S24 derivate, ∷ *P_TEF_-NIT1-T_PET9_* | This study |
| S44 | S24 derivate, ∷ *P_TEF_-SPT10-T_PET9_* | This study |
| Δ*PDR12* | GS115 derivate, Δ*PDR12* | This study |
| Δ*War1* | GS115 derivate, Δ*War1* | This study |
| Δ*PDR12*Δ*War1* | GS115 derivate, Δ*PDR12*Δ*War1* | This study |
| Δ*12*Δ*C4R916* | GS115 derivate, Δ*PDR**12*Δ*C4R916* | This study |
| Δ*12*Δ*YDR541C* | GS115 derivate, Δ*PDR12*Δ*YDR541C* | This study |
| Δ*12*Δ*C4R2I5* | GS115 derivate, Δ*PDR12*Δ*C4R2I5* | This study |
| S45 | S43 derivate, Δ*PDR12*Δ*C4R2I5* | This study |

**Table S3.** Plasmids used in this study.

| Names | Characteristics | |
| --- | --- | --- |
| Plasmids |  |  |
| pCas | P3.5k ori, Kan, P_cas_-cas9, P_HIS_-His4, P_HTX1_-Int10-N20 |  |
| PPK2 | pVS1 StaA, Kan | This study |
| pCas-Int10 | P3.5k ori, Kan, P_cas_-cas9, PHIS-His4, PHTX1-Int15-N20 | This study |
| pCas-Int15 | P3.5k ori, Kan, P_cas_-cas9, PHIS-His4, PHTX1-Int15-N20 | This study |
| pCas-Int21 | P3.5k ori, Kan, P_cas_-cas9, PHIS-His4, PHTX1-Int21-N20 | This study |
| pCas-Int6 | P3.5k ori, Kan, P_cas_-cas9, PHIS-His4, PHTX1-Int6-N20 | This study |
| pCas-Int18 | P3.5k ori, Kan, P_cas_-cas9, PHIS-His4, PHTX1-Int18-N20 | This study |
| pCas-PDR12 | P3.5k ori, Kan, P_cas_-cas9, PHIS-His4, PHTX1-PDR12-N20 | This study |
| pCas-WAR1 | P3.5k ori, Kan, P_cas_-cas9, PHIS-His4, PHTX1-WAR1-N20 | This study |
| pCas-C4R916 | P3.5k ori, Kan, P_cas_-cas9, PHIS-His4, PHTX1-C4R916-N20 | This study |
| pCas-YDR541C | P3.5k ori, Kan, P_cas_-cas9, PHIS-His4, PHTX1-YDR541C-N20 | This study |
| pCas-C4R2I5 | P3.5k ori, Kan, P_cas_-cas9, PHIS-His4, PHTX1-C4R2I5-N20 | This study |
| PPK2-PCL1 | pVS1 StaA, Kan, TEF1p, PCL1 | This study |
| PPK2-C4R6P5 | pVS1 StaA, Kan, TEF1p, C4R6P5 | This study |
| PPK2-C4QZ73 | pVS1 StaA, Kan, TEF1p, C4QZ73 | This study |
| PPK2-YurZ | pVS1 StaA, Kan, TEF1p, YurZ | This study |
| PPK2-C4R1W3 | pVS1 StaA, Kan, TEF1p, C4R1W3 | This study |
| PPK2-GPR1 | pVS1 StaA, Kan, TEF1p, GPR1 | This study |
| PPK2-Tpo1 | pVS1 StaA, Kan, TEF1p, Tpo1 | This study |
| PPK2-MCH2 | pVS1 StaA, Kan, TEF1p, MCH2 | This study |
| PPK2-ATG1 | pVS1 StaA, Kan, TEF1p, ATG1 | This study |
| PPK2-YBL055C | pVS1 StaA, Kan, TEF1p, YBL055C | This study |
| PPK2-WYB5 | pVS1 StaA, Kan, TEF1p, WYB5 | This study |
| PPK2-ARG1 | pVS1 StaA, Kan, TEF1p, ARG1 | This study |
| PPK2-ARG3 | pVS1 StaA, Kan, TEF1p, ARG3 | This study |
| PPK2-NIT1 | pVS1 StaA, Kan, TEF1p, NIT1 | This study |
| PPK2-SPT10 | pVS1 StaA, Kan, TEF1p, SPT10 | This study |
| PPK2-LYS1 | pVS1 StaA, Kan, TEF1p, LYS1 | This study |
| PPK2-AVT1 | pVS1 StaA, Kan, GAP1p, AVT1 | This study |
| PPK2-LYP1 | pVS1 StaA, Kan, GAP1p, LYP1 | This study |
| PPK2-PUT4 | pVS1 StaA, Kan, GAP1p, PUT4 | This study |
| PPK2-BUL1 | pVS1 StaA, Kan, GAP1p, BUL1 | This study |
| PPK2-ARO9 | pVS1 StaA, Kan, GCW14p, ARO9 | This study |
| PPK2-ARO8 | pVS1 StaA, Kan, GCW14p, ARO8 | This study |
| PPK2-HIS5 | pVS1 StaA, Kan, GCW14p, HIS5 | This study |
| PPK2-BAT1 | pVS1 StaA, Kan, GCW14p, BAT1 | This study |
| PPK2-AAT2 | pVS1 StaA, Kan, GCW14p, AAT2 | This study |
| PPK2-CAD1 | pVS1 StaA, Kan, PGK1p, CAD1 | This study |
| PPK2-CAD3 | pVS1 StaA, Kan, PGK1p, CAD3 | This study |
| PPK2-ADH2 | pVS1 StaA, Kan, PGK1p, ADH2 | This study |
| PPK2-ALD4 | pVS1 StaA, Kan, PGK1p, ALD4 | This study |

**Table S4**. gRNA used in this study

| primers | Primer sequences (5’-3’) | |
| --- | --- | --- |
| N20-Int10 | AATTACTTCGGGAATAATGG | This study |
| N20-Int15 | GACTCTCCACAAGTTAACCA | This study |
| N20-Int21 | GCACCATCTGGATAGCATTG | This study |
| N20-Int6 | CAACTCGAATTATAGTGGCG | This study |
| N20-Int18 | TACATGGAATAGGGTCACGT | This study |
| N20-PDR12 | ACTGCTGTTACTGATCCAAG | This study |
| N20-War1 | GTAGATGTGAACGATCCTGG | This study |
| N20-ESBP6 | CAGATGGGGGAATCACTGCG | This study |
| N20-TUB2 | GACGATGAAGGAAACTACGT | This study |
| N20-TUB1 | TTGCTGGGAGCTGTACACAA | This study |
| N20-FTR1 | GAAGGGTAAAAACAAGAAGG | This study |
| N20-C4R3M0 | GCATTTGAAAAATCTCACGG | This study |
| N20-C4R296 | TGGAATGACATAGATAGTGG | This study |
| N20-FEN2 | ATAGAATCATTCCTGCCCTG | This study |
| N20-ERG2 | CTCCAGGAATGCATAGACGA | This study |
| N20-C4R3C4 | ACTCAAAACTTGAACACCAG | This study |
| N20-HHT1 | CAAACAGCAAGAAAATCCAC | This study |
| N20-SLT2 | ATATTGGCCGAACTCTTAGG | This study |
| N20-OPI10 | GGGCATCTATAAACCCACTG | This study |
| N20-GGT | TCGACAATCAAAACTCAGAG | This study |
| N20-ODC1 | ATGAACATTCTGGATGTAGG | This study |
| N20-URE2 | TAGGATCCCAACTTTGACGG | This study |
| N20-RRM2 | ACTGCTGAGGAAATAGACCT | This study |
| N20-SSA1 | GCCAGATTCGAGGAACTGTG | This study |
| N20-HtpG | AATCCAGTTAGTCGTCACCA | This study |
| N20-KAR2 | AGAAAGCAGAGAAACATCGA | This study |
| N20-YDJ1 | GGAGAAGGTGATATAATCAG | This study |
| N20-FES1 | TTATCCAAAGATGTCAACAG | This study |
| N20-SSA4 | TTAGAATGTTGTGCTCACCG | This study |
| N20-CNE1 | GGGTCTGGTTGTGAAATCTG | This study |
| N20-SCJ1 | AAAGTACTCATCCTCCCCAA | This study |
| N20-DSK2 | GTTCAAAGGAAAGATTGCCG | This study |
| N20-HLJ1 | GTTGTCGATATCTTCAAACG | This study |
| N20-SLC1 | GAATTTCTGCAAAACAACCA | This study |
| N20-C4R916 | TCAGACTACGAGCTGATTTG | This study |
| N20-YPS1 | ATGGTGTTGTGGATTCATTG | This study |
| N20-DFG5 | GGGTTTATCAACGACCAAGG | This study |
| N20-PXMP2 | GCTCACAAAGAGTGTTACCG | This study |
| N20-PDR12 | GCATGGGATAATGCTACCAG | This study |
| N20-PRY2 | TGGAGCAGCTGAGACAACGG | This study |
| N20-SCW4 | TTGACCGATTTGGGAAACAG | This study |
| N20-SPE3 | TGTTCTCTGTGATATTGACG | This study |
| N20-YDR541C | AATATCTGGAACAAGCACAT | This study |
| N20-C4R1W3 | TAACGAACGAATCTCAGTTG | This study |
| N20-C4R2I5 | GAGTGCCGAAAAATTTACAG | This study |
| N20-HHF2 | GACTTCTTCGTAGATCAAAG | This study |
| N20-ATP15 | AGAGTTGCTGCTGAAAAGAG | This study |
| N20-C4QZX3 | TAACAGATGAACTCTCAGCA | This study |
| N20-C4QZR6 | GTTAGTGTATGAAAAACTGG | This study |
| N20-SSA2 | GAAGAGTTCAGCAACCAAGG | This study |
| N20-NIT1 | AAGCACACTTTACTGCACAA | This study |
| N20-C4QXY1 | AAGATAACAGCAAAGTACGG | This study |
| N20-RPL37 | AAGATGAGATCTTACAACTG | This study |
| N20-C4QW22 | ATTGCTATGGCAGACTTGTG | This study |
| N20-RPP1A | AAGGGCGGCGTATGATAAAG | This study |
| N20-C4R6T1 | TTATGGTGTCAAAGTCTACC | This study |
| N20-FDH1 | CCATCCAGCCTACATCACGA | This study |
| N20-YDR541C | AGAAGCAGTATGAAGAACAT | This study |
| N20-Rpl43A | TAATAGACAACTGTTAAAGG | This study |
| N20-SSA3 | TTTCAAAGTTATCAACAAGG | This study |

**Table S5** 33 differential target genes identified in this study

| Gene | | Phe-0h_FPKM | Phe-4h_FPKM | Phe-12h_FPKM | Phe-24h_FPKM |
| --- | --- | --- | --- | --- | --- |
| KDC1 | PAS_chr3_0095 | 133.84 | 1694.18 | 555.42 | 795.35 |
| KDC2 | PAS_chr4_0314 | 182.15 | 14688.03 | 2018.93 | 1427.02 |
| AVT1 | PAS_chr3_0226 | 51.60 | 1061.78 | 353.04 | 149.60 |
| LYP1 | PAS_chr1-1_0341 | 96.58 | 77.37 | 1097.51 | 740.93 |
| PUT4 | PAS_chr2-1_0659 | 55.56 | 303.87 | 208.07 | 134.76 |
| GAP5 | PAS_chr1-4_0479 | 274.11 | 184.93 | 27.95 | 17.98 |
| GAP1 | PAS_chr1-1_0030 | 51.08 | 110.94 | 149.38 | 452.12 |
| C4R341 | PAS_chr2-2_0022 | 71.19 | 4694.55 | 2453.12 | 819.78 |
| ARO9 | PAS_chr4_0147 | 49.94 | 8906.13 | 5008.69 | 10061.46 |
| AR08 | PAS_chr1-4_0608 | 279.74 | 927.91 | 513.34 | 281.66 |
| HIS5 | PAS_chr2-1_0684 | 72.23 | 1045.08 | 584.73 | 268.15 |
| BAT1 | PAS_chr4_0248 | 918.79 | 10061.83 | 4776.00 | 2158.79 |
| AAT2 | PAS_chr4_0974 | 178.67 | 1245.65 | 486.71 | 160.53 |
| CAD1 | PAS_chr3_0006 | 195.34 | 488.35 | 884.20 | 1008.69 |
| CAD3 | PAS_chr2-1_0472 | 7122.26 | 1172.07 | 3823.79 | 3757.05 |
| ADH2 | PAS_chr2-1_0313 | 2925.31 | 7686.21 | 2238.54 | 1705.02 |
| ALD4 | PAS_chr2-1_0853 | 178.24 | 64.81 | 380.64 | 700.40 |
| PCL1 | PAS_chr1-1_0097 | 70.62 | 8841.44 | 4059.05 | 1862.91 |
| C4R6P5 | PAS_chr4_0042 | 62.36 | 5635.88 | 7123.09 | 2476.05 |
| C4QZ73 | PAS_FragB_0015 | 74.43 | 4261.22 | 1988.10 | 640.65 |
| YurZ | PAS_chr3_0355 | 42.20 | 1990.74 | 289.84 | 124.40 |
| C4R1W3 | PAS_chr2-2_0422 | 23.02 | 1063.65 | 81.09 | 45.22 |
| GPR1 | PAS_chr1-1_0378 | 21.75 | 968.36 | 213.45 | 239.99 |
| Tpo1 | PAS_chr1-3_0215 | 21.00 | 923.90 | 696.76 | 254.54 |
| MCH2 | PAS_chr1-4_0132 | 49.53 | 2063.14 | 413.38 | 123.62 |
| ATG1 | PAS_chr2-1_0641 | 20.93 | 579.83 | 373.76 | 270.19 |
| YBL055C | PAS_chr2-2_0215 | 44.39 | 1196.90 | 411.70 | 143.13 |
| WYB | PAS_chr4_0146 | 58.80 | 5469.71 | 2129.06 | 639.87 |
| ARG1 | PAS_chr3_0216 | 151.44 | 3763.12 | 1803.88 | 679.47 |
| ARG3 | PAS_chr3_0623 | 15.96 | 467.95 | 229.00 | 85.85 |
| NIT1 | PAS_chr2-1_0037 | 27.53 | 4168.19 | 1960.66 | 467.29 |
| SPT10 | PAS_chr2-2_0140 | 21.61 | 557.00 | 300.31 | 263.31 |
| LYS1 | PAS_chr1-1_0447 | 191.36 | 4531.85 | 2791.65 | 1504.52 |

**Table S6**: Differentially expressed ABC transporter genes at the transcriptional level.

| Gene | | 0g/L 2-PE FPKM | 1.5g/L 2-PE FPKM | 2.0g/L 2-PE FPKM |
| --- | --- | --- | --- | --- |
| PAS_chr1-1-0398 | PDR5 | 221.43 | 589.45 | 1360.30 |
| PAS_chr2-2-0299 | PDR10 | 86.54 | 254.74 | 187.03 |
| PAS_chr2-1-0190 | 210190 | 23.31 | 47.91 | 47.19 |
| PAS-chr4-0186 | YOR1 | 27.30 | 3.61 | 7.91 |
| PAS-chr2-2-0272 | PXA2 | 77.58 | 33.01 | 35.43 |
| PAS-FragB-0065 | BOO65 | 195.40 | 41.17 | 40.75 |
| PAS-chr4-0832 | PDR12 | 67.31 | 707.13 | 1056.43 |

**Supplementary Figures**

**c
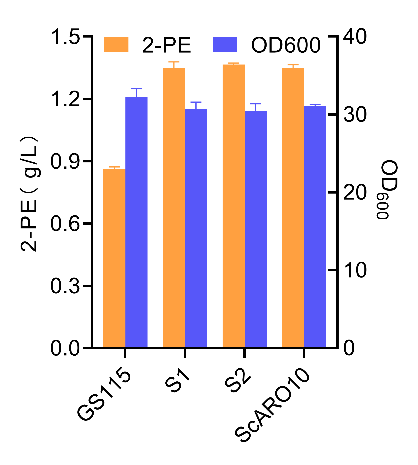
**

**Figure S1.** Fermentation yield of 2-PE obtained by overexpressing the phenylalanine decarboxylase strain of *S. cerevisiae*
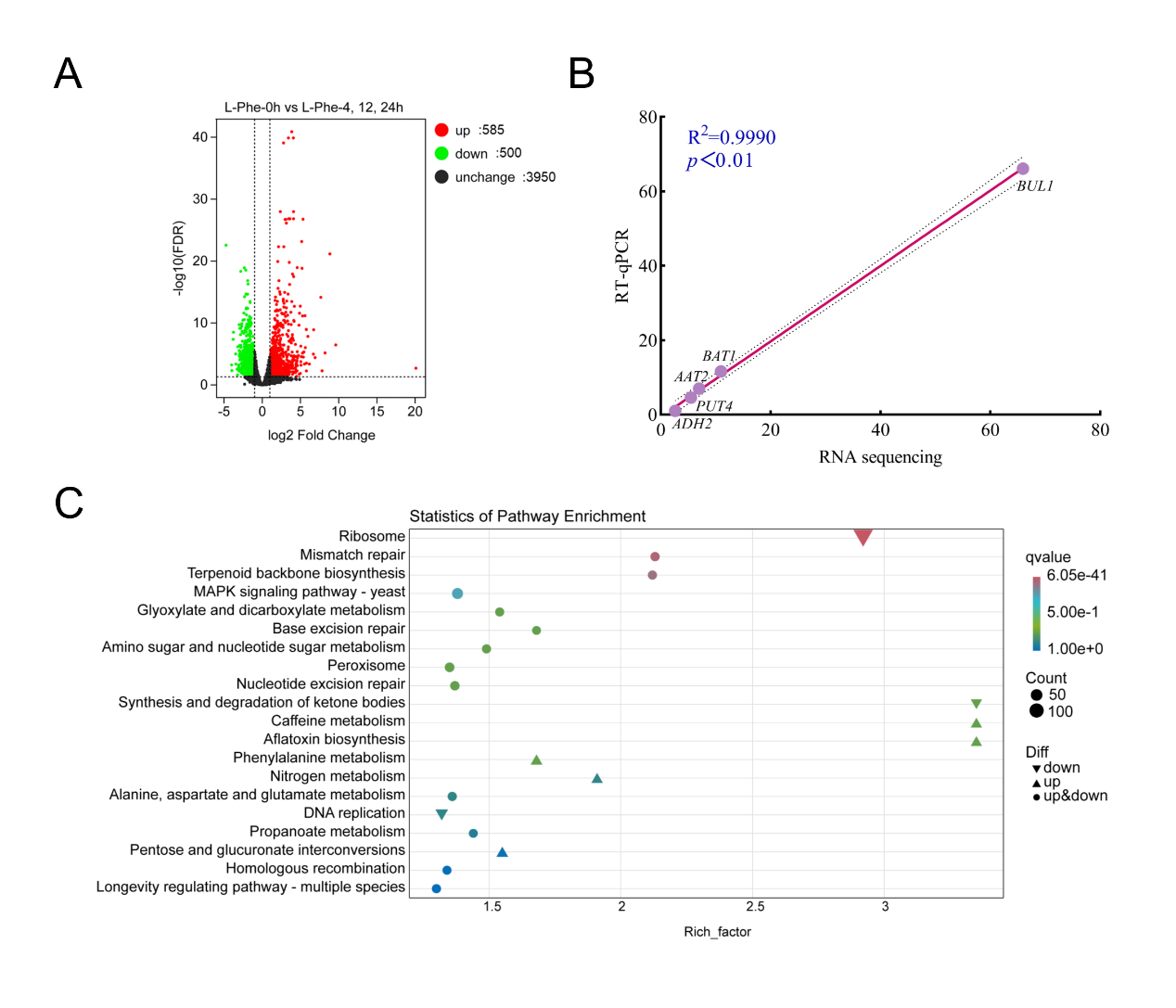


**Figure S2.** Transcriptome analysis of strains without L-Phe feeding and with L-Phe feeding for 4, 12 and 24 h. (A) Volcano maps of differentially expressed genes in strains without L-Phe feeding and L-Phe feeding for 4,12 and 24 hours. (B) Validation of the correlation between RT-qPCR and transcriptome data. (C) KEGG enrichment bubble diagram of differential genes in strains without L-Phe feeding and L-Phe feeding for 4,12 and 24 hours.


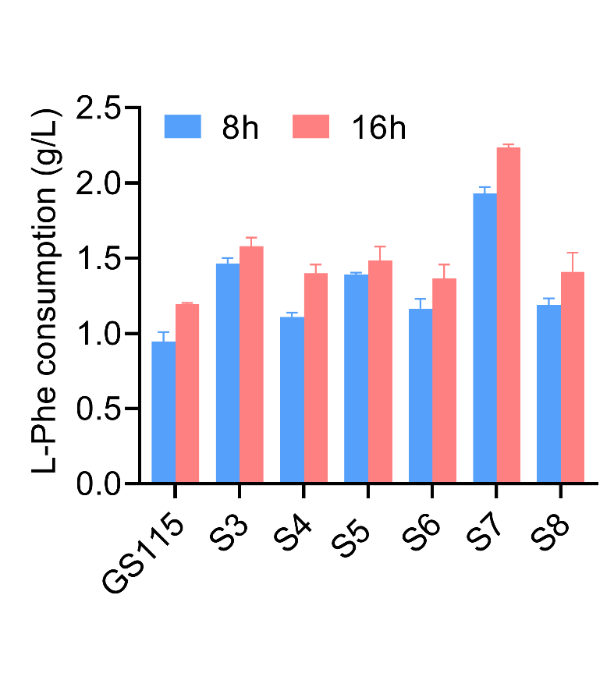


**Figure S3.** The consumption of L-Phe in the transporter-overexpressing strain at 8 h and 16 h.


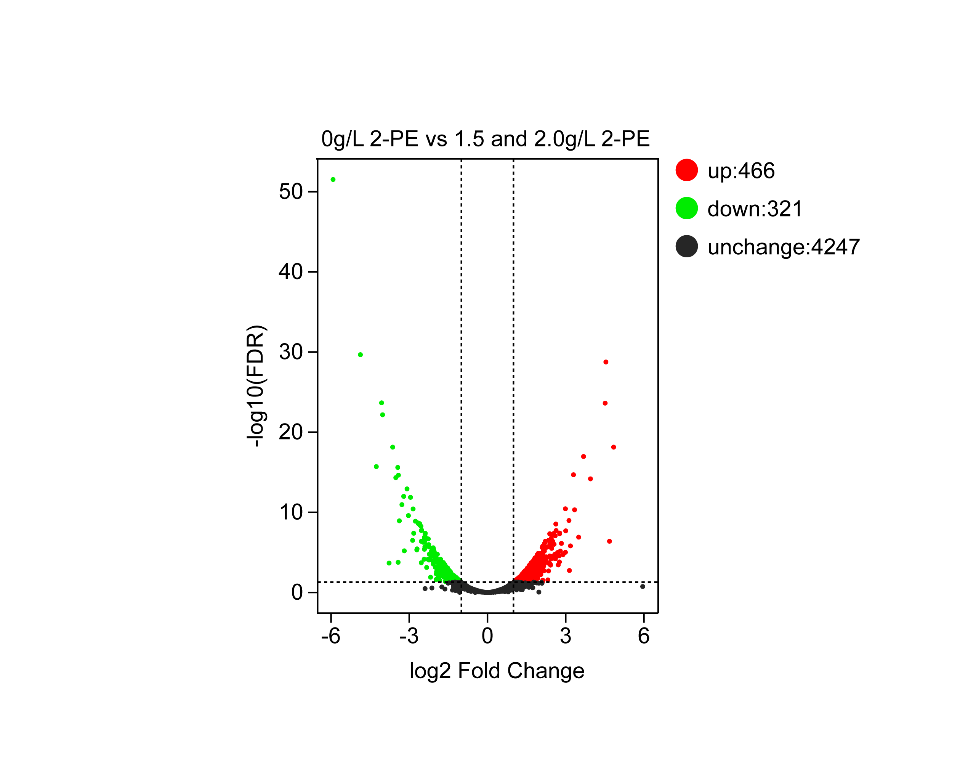


**Figure S4.** Volcano plot of differentially expressed genes in the strains after 4 h of 0 g/L 2-PE stress and 4 h of 1.5 g/L 2-PE and 2 g/L 2-PE stress.


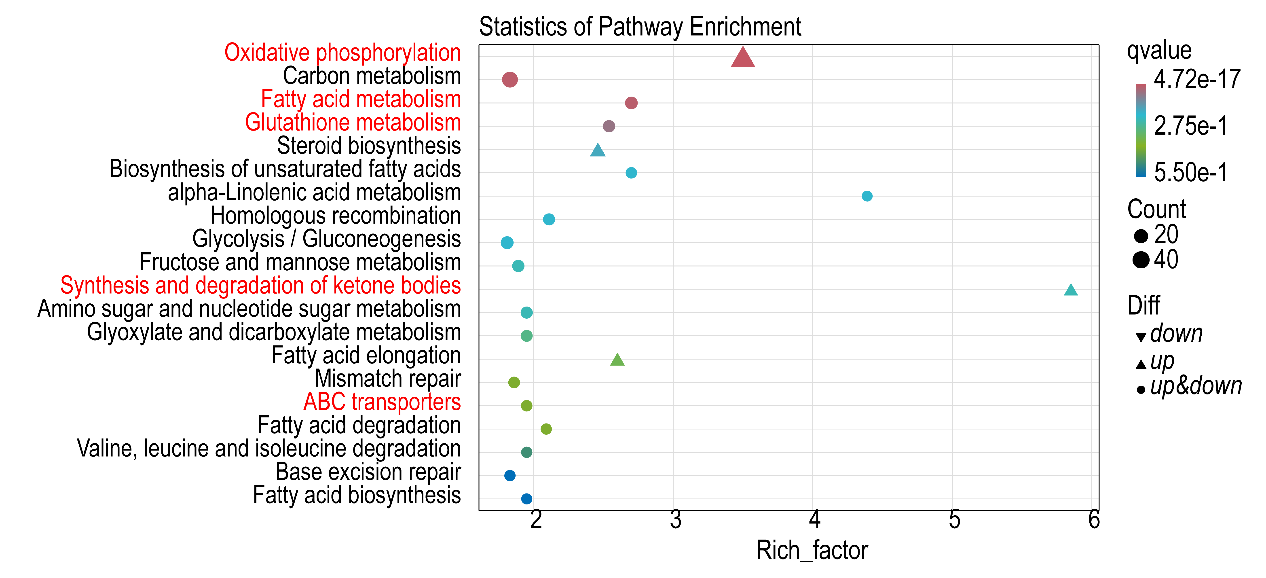


**Figure S5.** KEGG enrichment bubble diagram of the DEGs in the strains after 4 h of 0 g/L 2-PE, 1.5 g/L 2-PE or 2 g/L 2-PE stress.


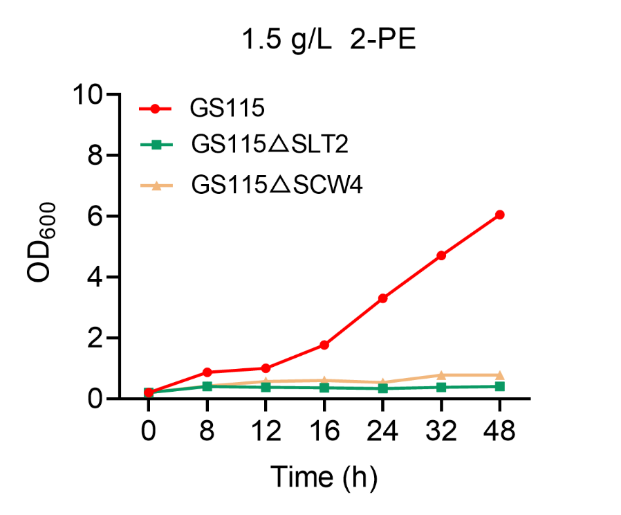


**Figure S6.** Growth curve of the recombinant strain under 1.5 g/L 2-PE stress.

(1) Kong, S.; Pan, H.; Liu, X.; Li, X.; Guo, D., De novo biosynthesis of 2-phenylethanol in engineered Pichia pastoris. *Enzyme Microb Technol* **2020,** *133*, 109459).

(2) Hassing, E.-J.; de Groot, P. A.; Marquenie, V. R.; Pronk, J. T.; Daran, J.-M. G., Connecting central carbon and aromatic amino acid metabolisms to improve de novo 2-phenylethanol production in Saccharomyces cerevisiae. *Metabolic Engineering* **2019,** (56), 165-180.

(3) Romagnoli, G.; Luttik, M. A. H.; Kötter, P.; Pronk, J. T.; Daran, J.-M., Substrate specificity of thiamine pyrophosphate-dependent 2-oxo-acid decarboxylases in Saccharomyces cerevisiae. *Appl Environ Microbiol* **2012,** *78* (21), 7538-7548.

(4) Gu, Y.; Ma, J.; Zhu, Y.; Ding, X.; Xu, P., Engineering Yarrowia lipolytica as a Chassis for De Novo Synthesis of Five Aromatic-Derived Natural Products and Chemicals. *ACS Synthetic Biology* **2020,** *9* (8), 2096-2106.

(5) Rajkumar, A. S.; Morrissey, J. P., Rational engineering of Kluyveromyces marxianus to create a chassis for the production of aromatic products. *Microbial Cell Factories* **2020,** *19* (1), 207.

(6) Li, M.; Lang, X.; Moran Cabrera, M.; De Keyser, S.; Sun, X.; Da Silva, N.; Wheeldon, I., CRISPR-mediated multigene integration enables Shikimate pathway refactoring for enhanced 2-phenylethanol biosynthesis in Kluyveromyces marxianus. *Biotechnol Biofuels* **2021,** *14* (1), 3.

(7) Guo, D.; Zhang, L.; Kong, S.; Liu, Z.; Li, X.; Pan, H., Metabolic Engineering of Escherichia coli for Production of 2-Phenylethanol and 2-Phenylethyl Acetate from Glucose. *Journal of Agricultural and Food Chemistry* **2018,** *66* (23), 5886-5891.

(8) Zhan, Y.; Shi, J.; Xiao, Y.; Zhou, F.; Wang, H.; Xu, H.; Li, Z.; Yang, S.; Cai, D.; Chen, S., Multilevel metabolic engineering of Bacillus licheniformis for de novo biosynthesis of 2-phenylethanol. *Metabolic Engineering* **2022,** *70*, 43-54.

(9) Gu, Y.; Ma, J.; Zhu, Y.; Xu, P., Refactoring Ehrlich Pathway for High-Yield 2-Phenylethanol Production in Yarrowia lipolytica. *ACS Synthetic Biology* **2020,** *9* (3), 623-633.

(10) Wang, Z.; Jiang, M.; Guo, X.; Liu, Z.; He, X., Reconstruction of metabolic module with improved promoter strength increases the productivity of 2-phenylethanol in Saccharomyces cerevisiae. *Microb Cell Fact* **2018,** *17* (1), 60.

(11) Zhao, Y.; Li, S.; Shu, Q.; Yang, X.; Deng, Y., Highly efficient production of 2-phenylethanol by wild-type Saccharomyces bayanus strain. *Bioresource Technology* **2024**, 130867.

(12) Wang, Z.; Bai, X.; Guo, X.; He, X., Regulation of crucial enzymes and transcription factors on 2-phenylethanol biosynthesis via Ehrlich pathway in Saccharomyces cerevisiae. *Journal of Industrial Microbiology & Biotechnology* **2017,** *44* (1), 129-139.

(13) Chen, X.; Wang, Z.; Guo, X.; Liu, S.; He, X., Regulation of general amino acid permeases Gap1p, GATA transcription factors Gln3p and Gat1p on 2-phenylethanol biosynthesis via Ehrlich pathway. *J Biotechnol* **2017,** *242*, 83-91.

(14) Yin, S.; Zhou, H.; Xiao, X.; Lang, T.; Liang, J.; Wang, C., Improving 2-phenylethanol production via Ehrlich pathway using genetic engineered Saccharomyces cerevisiae strains. *Curr Microbiol* **2015,** *70* (5), 762-767.

(15) Wang, Y.; Zhang, Z.; Lu, X.; Zong, H.; Zhuge, B., Genetic engineering of an industrial yeast Candida glycerinogenes for efficient production of 2-phenylethanol. *Applied Microbiology and Biotechnology* **2020,** *104* (24), 10481-10491.

(16) Zhan, Y.; Zhou, M.; Wang, H.; Chen, L.; Li, Z.; Cai, D.; Wen, Z.; Ma, X.; Chen, S., Efficient synthesis of 2-phenylethanol from L-phenylalanine by engineered Bacillus licheniformis using molasses as carbon source. *Appl Microbiol Biotechnol* **2020,** *104* (17), 7507-7520.
